# Non-destructive analyses of iron-polyphenolic complexes for reconstructing iron-gall inks historical recipes

**DOI:** 10.1038/s40494-025-01946-x

**Published:** 2025-07-28

**Authors:** Salvatore Caterino, Iulia-Maria Caniola, Katja Sterflinger, Marc Pignitter, Federica Cappa

**Affiliations:** 1 Institute of Natural Sciences and Technology in the Arts, Academy of Fine Arts of Vienna, Vienna, Austria; 2 Institute of Physiological Chemistry, Faculty of Chemistry—University of Vienna, Vienna, Austria

## Abstract

Iron-gall inks (IGI) show considerable variability due to the wide range of historical recipes used in their preparation. Previous research has explored how factors such as pH and iron concentration affect IGI structure and how to detect these changes. This study focuses on variability induced by different tannins interacting with iron. Iron–polyphenolic complexes were systematically synthesized using both commercial tannins and oak gall extracts prepared following historical recipes. All starting materials were characterized via spectrophotometric assays and, for commercial tannins, infrared spectroscopy. The resulting complexes were analysed using Raman, IR, and Electron Paramagnetic Resonance spectroscopies to build a reference dataset. This enabled the identification of spectral markers offering insight into the materials used in IGI production through a non-destructive, multi-analytical approach. Finally, the method was applied to the “Black Hours” manuscript (property of the Austrian National Library) to investigate its ink composition, highlighting both the strengths and limits of the applied techniques.

## Introduction

Iron-gall inks (IGI) are among the most used writing materials in ancient times. They were historically produced starting from three main components: a botanical extract (sometimes also defined as an infusion) rich in polyphenols, and in particular in tannins, a source of iron and an organic binder^1–5^. The polyphenols contained in the extract are capable of effectively bind iron cations, forming insoluble particles of metal-polyphenolic networks characterized by a dark-bluish color. The chemistry behind the production of this kind of inks is far from being simple. The process is in fact, strongly affected by several factors, including the type of polyphenolic compounds present in the extract, the pH conditions, and the ratio between polyphenols and iron cations^1,6–10^. Moreover, due to their large diffusion^1^, an impressive number of historical recipes for their preparation have been recorded, resulting in a great variability of inks’ chemical composition. This variability can be attributed to several factors, including the origin and quality of the raw materials, the specific historical recipes employed, which dictate the proportions of ingredients and the processing methods used, and, finally, the type and quantity of additives incorporated^1,2,4,11–14^.

**Fig. 1:**
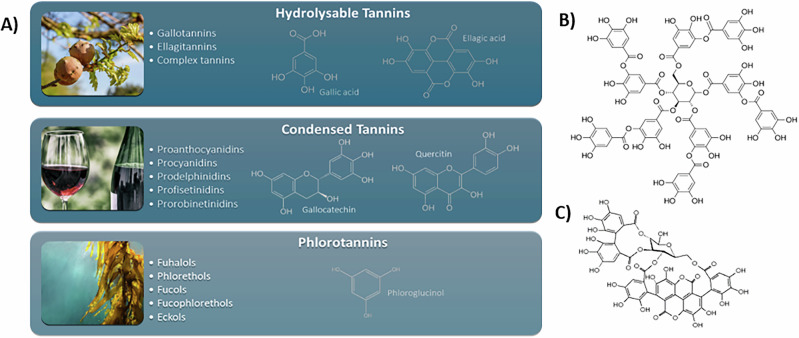
Classification of natural tannins. **A** Summary of the main classes and subclasses of natural tannins. **B** Molecular structure of tannic acid (TA), one of the most abundant tannins present in oak-galls. **C** Molecular structure of punicalagin, an ellagitannin isolated from pomegranate.

Aleppo oak galls (OG) represent one of the most commonly mentioned botanical matrices for extract preparation^1–3^. However, the incorporation of additives such as wine, pomegranate peels, and chestnuts enlarges the pool of polyphenols capable of interacting with iron ions^1,8,12^. Such interactions are strongly influenced by the structural features of the polyphenols, including the number and type of binding sites and their chemical environment^4,5,11,12^, which, in turn, dictate the final properties of the ink and its degradation patterns. The main constituents of the polyphenolic pool present in these extracts are tannins. Tannins constitute a large class of biomolecules acting within the plant as secondary metabolites (or anti-nutrients) due to their good affinity with most of the biomacromolecules^15,16^. They can generally be easily extracted with hot water from the botanical matrices. This aspect contributes to their large use for a great variety of applications since ancient times, when they were used as tanning agents in the leathering process^17,18^. From a chemical perspective, this large group of molecules is divided into classes and subclasses (Fig. 1). Hydrolysable tannins (HT) are characterized by a central sugar esterified with gallic acid (GA) or other polyphenolic compounds. Depending on the esterifying polyphenols, HT are categorized into three primary subclasses: gallotannins (GT), esterified exclusively with GA; ellagitannins (ET), esterified with one or more units of ellagic acid (EA), a GA derivate formed through oxidative coupling; and complex tannins (CoT), involving one or more flavonoid units^19–23^. Condensed tannins (CT) instead are composed of flavan-3-ol units linked via C-C bonds, and further subdivided based on the type of linkage and structural variations of the flavanol units, particularly the hydroxylation patterns of the aromatic rings and the stereochemistry of the aliphatic hydroxyl groups^19,24,25^. Phlorotannins (PT), primarily synthesized by brown algae, are less frequently encountered and thus are sometimes omitted from tannin classifications^19,21,26^.

To date, the distinction among various IGI has been mainly focused on their “inorganic component”. The elements of variability previously listed do, in fact, often result in qualitative and quantitative differences in the metals present in the final inks, easily detectable with non-invasive techniques such as X-ray Fluorescence (XRF)^1,27–29^. Several in-depth studies of the organic components of IGI have been carried out to access this variability^2,30,31^. However, these studies rely on invasive approaches such as high performance liquid chromatography (HPLC) coupled with mass spectrometry, using different configurations like simple electrospray ionization and an ion trap quadrupole (ESI-Q) or tandem mass spectrometry such as electrospray ionization quadrupole time-of-flight (ESI-Q-ToF)^2,14,30,32^. While on one hand these approaches enable the obtainment of accurate qualitative and quantitative results, on the other hand they are destructive and may require time-consuming sample preparation^30^. These limitations could reduce the applicability of such methodologies for characterizing real manuscript samples. Therefore, this research addresses the challenge of the differentiation of IGIs from the perspective of the organic component, with a specific focus on the polyphenolic fraction, through non-destructive approaches.

This study employs Raman, Infrared (IR), and Electron Paramagnetic Resonance (EPR) spectroscopies to characterize iron-polyphenolic complexes prepared using commercial tannins and laboratory-prepared oak-gall extracts based on adapted historical recipes as sources of polyphenols. By adopting a systematic and multi-analytical approach, the potential of these non-invasive techniques is explored to provide deeper insights into the organic components of ink, contributing to the refinement of previous research and the reconstruction of historical recipes^31,33,34^. This approach was finally applied to three small fragments from the Codex 1856, 15th-century manuscript preserved at the ÖNB, in order to evaluate the effectiveness of these techniques in identifying the materials employed in its manufacture^35^. Given the high artistic and historical significance of this unique object, all analyses were conducted solely on minute fragments (from 5 × 2 mm to 5 × 8 mm), not newly sampled, but made available for analytical purposes from material recovered during prior conservation treatments.

## Methods

### Preparation of oak gall (OG) extracts

Reconstructing historical recipes under laboratory conditions often requires time-consuming steps, including archival research, translation into modern terminology, and conversion of historical units^1,2^. These tasks are complicated by variations in terminology across sources and the inconsistent use of measurement units^1^. For these reasons, the preparation of OG extracts was based on historical recipes previously mentioned in other studies^1,2,7,30^. The initial recipe was selected for its simplicity, while the latter two were chosen for their incorporation of tannin-containing ingredients, namely wine and pomegranate peel.

A simple extract (OG-Ex) has been prepared following the optimized protocol described by *Caterino* et al., which is based on a historical recipe contained in the manuscript H490 of the Medical Faculty of Montpellier, referred to below as “Montpellier”^1–4^. 2.85 g of roughly crushed Aleppo OG (purchased from Kremer Pigmente®) were mixed with 100 mL of deionized water (35 mL per gram of OG). The system was kept under moderate stirring at room temperature (RT) for three days. The insoluble parts were then removed via Buchner filtration. The resulting clear solution was concentrated via rotary evaporation to reduce its volume to 50 mL.

Two extracts containing wine (OGWW-Ex and OGRW-Ex) were prepared following a systematic protocol based on a Spanish recipe from the Madrid area, here referred to as “Madrid”^1,2^. In this case, 5.55 g of crushed Aleppo OG were mixed with 100 mL of commercially available white wine (OGWW-Ex). The same ratio between wine and OG (18.2 mL of wine per gram of OG) has been used in the case of OGRW-Ex, in which a commercial red wine was used. The two systems were kept under moderate stirring for 9 days at RT. The insoluble parts were finally removed via filtration.

Finally, the last extract (OGP-Ex) was prepared using a protocol adapted from a Spanish recipe related to the area of Cordoba, referred to as “Cordoba”^1,2^. 3.82 g of crushed Aleppo OG was mixed with 100 mL of deionized water (26.2 mL per gram of sample). Pomegranate peel was added in the proportion of 0.5 g per gram of OG. The system was kept under moderate stirring for 8 days at RT. Finally, the insoluble parts were removed via filtration.

A part of the extract solutions was directly used for the preparation of iron-tannin complexes, while the remaining part was reserved for characterization. To obtain stock solutions from the OG extracts, an aliquot of the filtered extracts was centrifuged at 10,000 rpm for 10 min in order to remove the fine insoluble particles. Finally, 1 mL of the clear supernatant was diluted with deionized water in a 100 mL volumetric flask.

### Characterization of commercial tannins and tannin-rich oak gall extracts

For this study, together with the OG extracts, several commercial tannins, mainly produced for the oenological industry, have been selected (Table 1). Stock solutions of the selected tannins were prepared by dissolving about 100 mg of sample in 100 mL deionized water.

**Table 1:** Commercial tannins and OG extracts used in this research

Tannin	Description^a^	Abbreviation
Tan’Activ QBC -SilvaTeam	Condensed—profisetidinic	QBC_S
Tan’Activ GC -SilvaTeam	Hydrolysable—Gallotannins	GC_S
Tan’Activ C -SilvaTeam	Hydrolysable—Ellagitannins	C_S
Omnivin 10 R -Ajinomoto	Grape Seeds extract “mixture of proanthocyanidins”	O10R
Oak Extract—Figli Guido Lapi	Oak wood extract	OE_FGL
Tan’Activ T80 -SilvaTeam	Hydrolysable—Gallotannins	T80_S
Tannin Galalcool -Laffort	Hydrolysable—Gallotannins	TG_L
Tanal 02—Ajinomoto	Hydrolysable—Gallotannins	T02
Simple oak-gall extract (Montpellier)	Hydrolysable—Gallotannins	OG-Ex
Wine-containing oak gall extract (Madrid)	Hydrolysable + Condensed tannins	OGRW-Ex
Wine-containing oak gall extract (Madrid)	Hydrolysable tannins (mainly)	OGWW-Ex
Pomegranate-containing oak gall extract (Cordoba)	Hydrolisable—Gallotannins + Ellagitannins	OGP-Ex

^a^The short descriptions are based on specifications available on the websites or in the technical data sheets provided by the producers/providers of the commercial tannins and reflect the expected composition for laboratory-prepared extracts as reported in the literature.

The pH of both commercial tannins and extract solutions was measured using a pH meter (Horiba LAQUAtwin). For commercial tannins, pH measurements were taken from 1000 ppm stock solutions, whereas for OG extracts, the pH was measured directly from the filtered, undiluted solutions. Triplicate measurements were performed for all sample solutions.

The total phenolic content (TPC) was quantified using the Folin-Ciocâlteu (FC) UV-VIS spectrophotometric method^13^. A 5-point calibration curve was prepared dissolving 50 mg of GA (purity ≥ 98.5%, Sigma Aldrich) in 50 mL of deionized water and then operating serial dilutions to obtain GA standards at 4, 6, 8, 10, and 20 ppm. A 0.5 mL aliquot of each standard was mixed with 2.5 mL of Folin reagent (Folin-Ciocalteu’s phenol reagent, Sigma Aldrich) previously diluted 10 times and, after 2 min, 2 mL of a 75 mg/mL Na₂CO₃ (≥99.5% purity, Sigma Aldrich) solution was added. The solutions were heated at 50 °C for 5 min, and absorbance at 765 nm (A_765_) was measured using a Spectroquant® Prove-100 UV-VIS spectrophotometer.

The tannin and extract stock solutions were first diluted 100 times for tannins and 50 times for extract stock solutions, which had already undergone a 100 times dilution from the original filtered and centrifuged solution. The diluted solutions were then analysed using the FC protocol described above. The TPC of the samples was determined using the GA calibration curve and expressed as mg of GA equivalents (GA eq.) per 100 mg of sample in the case of commercial tannins and as mg of GA eq. per 1 mL of extract solution for OG extracts. All samples and GA standard solutions were prepared and tested in triplicate.

To determine the CT fraction instead, the Butanol-HCl assay was used^13,14^. The recent protocol reported in *Zhen* et al. was employed. Specifically, 0.5 mL of the stock solutions was added to 5 mL of an acidic ferrous solution prepared by dissolving 38.5 mg of FeSO_4_·7H_2_O in 250 mL mixture composed of 2 parts of HCl (37%, Emsure Supelco, Merck) and 3 parts of butanol (BuOH) (99.9% purity, Sigma Aldrich). The mixtures were heated in a water bath at 95 °C for 15 minutes and then allowed to cool at room temperature. The absorbance at 530 nm (A_530_) was read and all the measurements have been performed in triplicate. Since no calibration curve was used, the results are expressed as mg of cyanidin unit equivalents (Cya eq.) per 100 mg of sample for commercial tannins and as mg of Cya eq. per 1 mL of extract solution for OG extracts. These values were calculated using a molar extinction coefficient of 34’700 M^−1^cm^−1^.

For commercial tannins only, 10–20 mg of pure tannin or extract powder was pressed into 5 mm-diameter pellets and analysed using Infrared (IR) following the instrumental settings reported below.

**Fig. 2:**
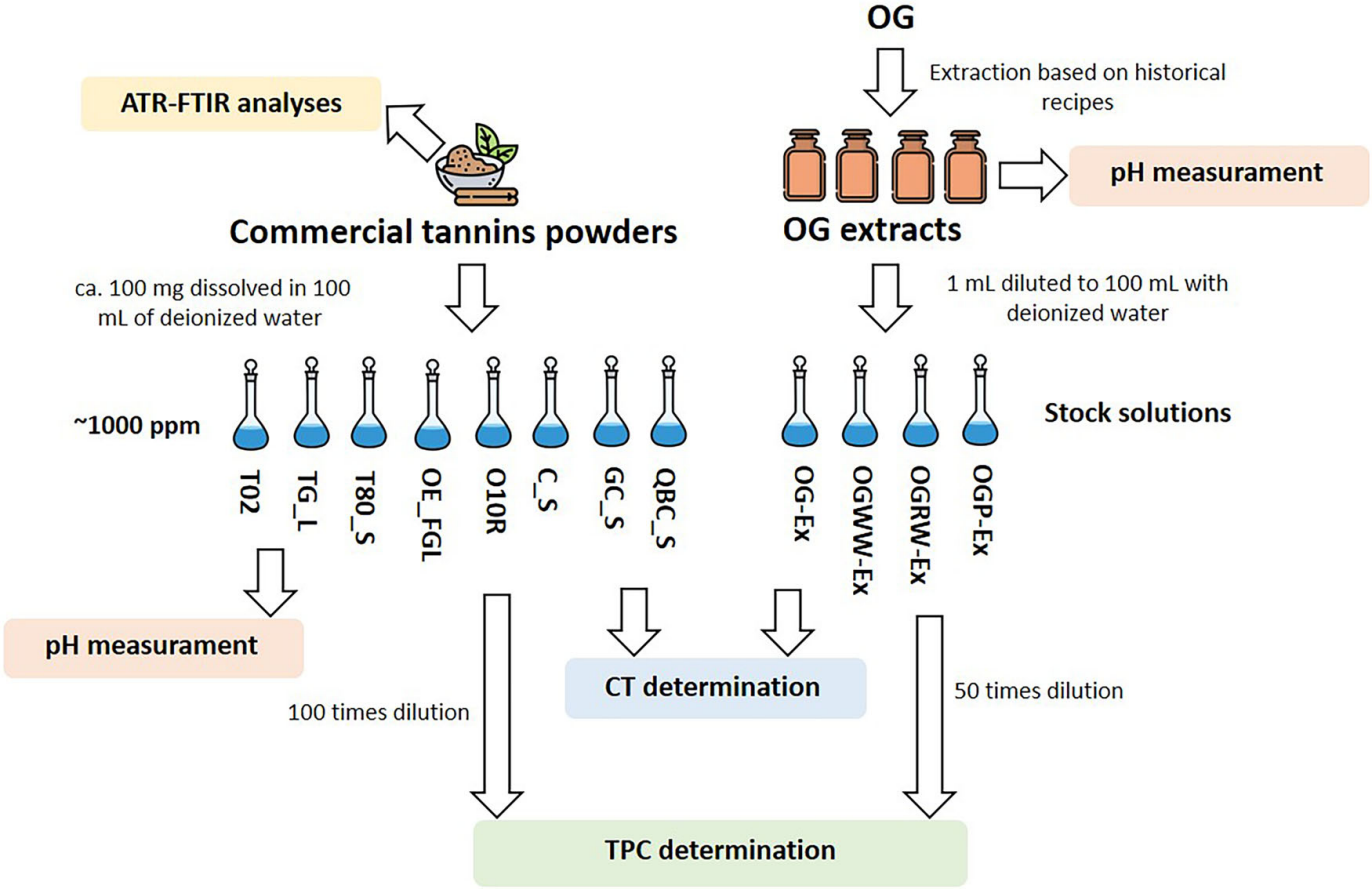
Methodological approach implemented for the commercial tannins’ and OG extracts’ characterization.

The overall methodological approach for tannins’ characterization is summarized in the scheme shown in Fig. 2.

### Preparation of iron-polyphenolic complexes using commercial tannins

Approximately 100 mg of tannin was completely dissolved in 100 mL of deionized water. 20 mL of each tannin solution was then mixed with around 1.00 mL of a freshly prepared 20 mg/mL solution of FeSO_4_·7H_2_O (99% purity, purchased from Chemsolute Th.Geyer®). The precise amount of iron solution to be added was calculated to achieve a 1:1 weight ratio between the iron salt and the tannin. The resulting solutions were kept in open Falcon tubes in a dark environment for at least 10 days to allow the oxidation of the initially formed soluble Fe(II)-tannin complexes into insoluble Fe(III)-tannin complexes. After the oxidation period, the precipitates have been separated from the solutions by centrifugation. At least 3 centrifugation-washing cycles turned out to be necessary (centrifugation: 14’500 rpm, 15 min). The precipitates have then been dried in an oven under mild conditions (24 h, 50 °C, 10% ventilation) and finally ground in an agate mortar. To improve the spectra acquisition procedure, about 15–20 mg of the fine powder has been pressed into 5 mm-diameter pellets using a pellet press die. All samples have been prepared in triplicate series.

### Preparation of iron-polyphenolic complexes using oak galls extracts

To better simulate historical ink preparation methods, the iron-polyphenolic complexes were formulated using the same ratio of OG to iron sulphate indicated in the three historical recipes selected for this study and mentioned in paragraph 1^2^. Specifically, 10 mL of each OG extract was mixed with a freshly prepared 250 mg/mL solution of FeSO₄·7H₂O as to have a weight ratio of OG: FeSO₄·7H₂O of 1.5:1 for OG-Ex, and 1:1 for OGWW-Ex, OGRW-Ex and OGP-Ex.

The table below (Table 2) summarizes the protocol used in the preparation of the extracts and the corresponding iron-polyphenolic complexes.

**Table 2:** Preparation protocols employed in the preparation of OG extracts and the related Fe-polyphenolic complexes

	Extract preparation				Fe-polyphenolic complex preparation		
Recipe	Ex. Solvent (mL)	OG (g)	Additive (g)	Procedure	OG : Fe^a^ (w:w)	g OG in 10 mL of extract^b^	mL of Fe solution to add^c^
Montpellier (OG-Ex)	35 (water)	1	/	Moderate stirring at RT for 3 days. Rotary evaporated to reduce the volume up to ¼. Filtration.	1.5 : 1	1.14	3.05
Madrid (OGWW-Ex and OGRW-Ex)	18 (wine)	1	/	Moderate stirring at RT for 9 days. Filtration.	1 : 1	0.56	2.24
Cordoba (OGP-Ex)	26.2 (water)	1	0.5 (pomegranate)	Moderate stirring at RT for 8 days. Filtration.	1 : 1	0.39	1.54

^a^OG : Fe denotes the mass ratio between the starting OG and FeSO₄·7H₂O.

^b^ The content in weight of OG in the extract is calculated considering the final volumes reported above.

^c^In this column, the mL of a 0.250 mg/mL solution of FeSO₄·7H₂O to be added to the 10 mL extract solution are reported.

The solutions were stored in open Falcon tubes in a dark environment for 10–15 days. The resulting precipitates were then isolated, dried, and pressed into pellets following the same protocol described in the previous paragraph. As in the previous case, all samples were prepared in triplicate.

### Raman characterization

Raman characterization has been performed directly on the sample pellets using the InVia-Qontor confocal Raman microscope from Renishaw®. The analyses on the commercial tannins and OG extracts-iron complexes have been carried out using the 785 nm diode laser line and using the following instrumental parameters: laser power 1% (maximum power of the laser: 300 mW), exposure time ranging from 5 to 10 s and 20 accumulations. A static grating scan modality was employed, minimizing the CCD detector area required and thus reducing the acquisition time, even though this approach limits the spectral range. Specifically, for all spectra, the centre was set at 1430 cm^−1^, with the lower and upper limits automatically adjusted to 946.93 cm^−1^ and 1880.93 cm^−1^, respectively, based on the 1200 l/mm grating (Table 3).

**Table 3:** Summary of the results related to the commercial tannins’ characterization

Tannin	Conc. stock solution^a^ (mg/mL)	pH	mg of GA eq. per 100 mg	mg of Cya eq. per 100 mg
QBC_S	1.04	4.85 ± 0.02	84.01 ± 2.35	54.75 ± 1.36
GC_S	1.00	4.09 ± 0.03	110.87 ± 1.87	0.41 ± 0.19
C_S	1.02	4.02 ± 0.01	72.73 ± 0.37	2.24 ± 0.21
O10R	1.06	3.79 ± 0.00	79.93 ± 1.97	108.97 ± 2.31
OE_FGL	1.02	3.96 ± 0.02	59.18 ± 1.22	2.90 ± 0.00
T80_S	1.07	3.37 ± 0.01	113.15 ± 1.41	1.14 ± 0.00
TG_L	1.01	3.93 ± 0.08	85.72 ± 0.98	1.28 ± 0.08
T02	1.10	4.14 ± 0.03	103.31 ± 0.72	0.16 ± 0.14

All reported values result from the average of the triplicate measurements, and the corresponding standard deviation (SD) is reported (in the format mean ± SD). See also tables T1.1-T1.3 in the Supporting Materials.

^a^For some of the samples, more than one stock solution was prepared. The reported concentration corresponds to the one at which the pH was measured. The concentrations of the solutions used for the Folin-Ciocalteu and BuOH/HCl assays are provided in the Supporting Materials (T1.2.2–T1.3).

The analyses on the manuscript’s fragments have been conducted using the same laser line (785 nm), but using the following parameters: laser power 50%, exposure time 10 s and 5 accumulations. In this case, an extended grating scan modality from 100 to 3000 cm^-1^ was employed (using the 1200 l/mm grating).

During spectral acquisition, a 50× magnification long working distance (LWD) objective was used, the pinhole was inserted, and high-confocality mode was selected. All spectra were processed using OPUS 7.5 software (Bruker). The processing steps included baseline correction (automatically performed using the concave rubber-band algorithm), smoothing (Savitzky–Golay algorithm, 21 points), and normalization (to the minimum and maximum values).

### IR characterization

Infrared (IR) characterization was conducted with a Bruker® Lumos II FT-IR microscope in Attenuated Total Reflectance (ATR) mode directly on the 5 mm-diameter pellets and the real samples. For each analysis, the focal plane array (FPA) 32 × 32 detector was used, with a scan time of 15 s for the mock-up pellets and 64 s for the manuscript’s fragment, the germanium crystal pressure set to ‘Low,’ and single-point measurement mode enabled. The FPA detector, combined with a ZnSe optic system, allows for signal acquisition over the spectral range of 5000–750 cm^−1^. With its 32 × 32 array of detector elements, the FPA captures a map of 1024 spectra in a single measurement. For each measurement, 4–6 spectra were extracted based on signal quality within the map and then processed using the OPUS 7.5 software (Bruker).

### EPR characterization

EPR characterization was performed using a Bruker Elexsys E 500 X-band spectrometer (∼9.75 GHz) equipped with a high-sensitivity SHQE1119 cavity (Bruker Biospin GmbH). Measurements were acquired in continuous wave (CW) mode at room temperature (RT). Each pellet used in the previous characterizations was split into two, with one portion directly placed in quartz EPR tubes for analysis.

The manuscript’s fragments (Fig. F.5 in Supporting Materials) were similarly inserted into EPR tubes for measurement. Acquisition parameters were set as follows: microwave power at 20 mW with 10 dB attenuation; modulation amplitude at 8 G; modulation frequency at 100 kHz; magnetic field centered at 3500 G with a sweep width of 6000 G. Three scans per measurement were acquired, each lasting 90 s (conversion time: 87.89 ms; RC time constant automatically set to 1/4 of the conversion time). Instrumental tuning was automatically performed via Xepr Software (Bruker). Spectra were processed with Origin2018. No baseline correction was applied. Signal intensities were normalized by sample weight, and field corrections were applied based on the tuning field of each sample.

## Results

### Commercial tannins and oak-gall extracts characterization

Previous studies demonstrated the importance of the pH condition prior to the iron addition in the formation process^7^. The tested samples shown pH values in the range of 2.25–4.85. In these conditions a 1-to-1 iron to ligand stoichiometric ratio is expected in the resulting iron-polyphenolic complexes^6,7,36^.

**Fig. 3:**
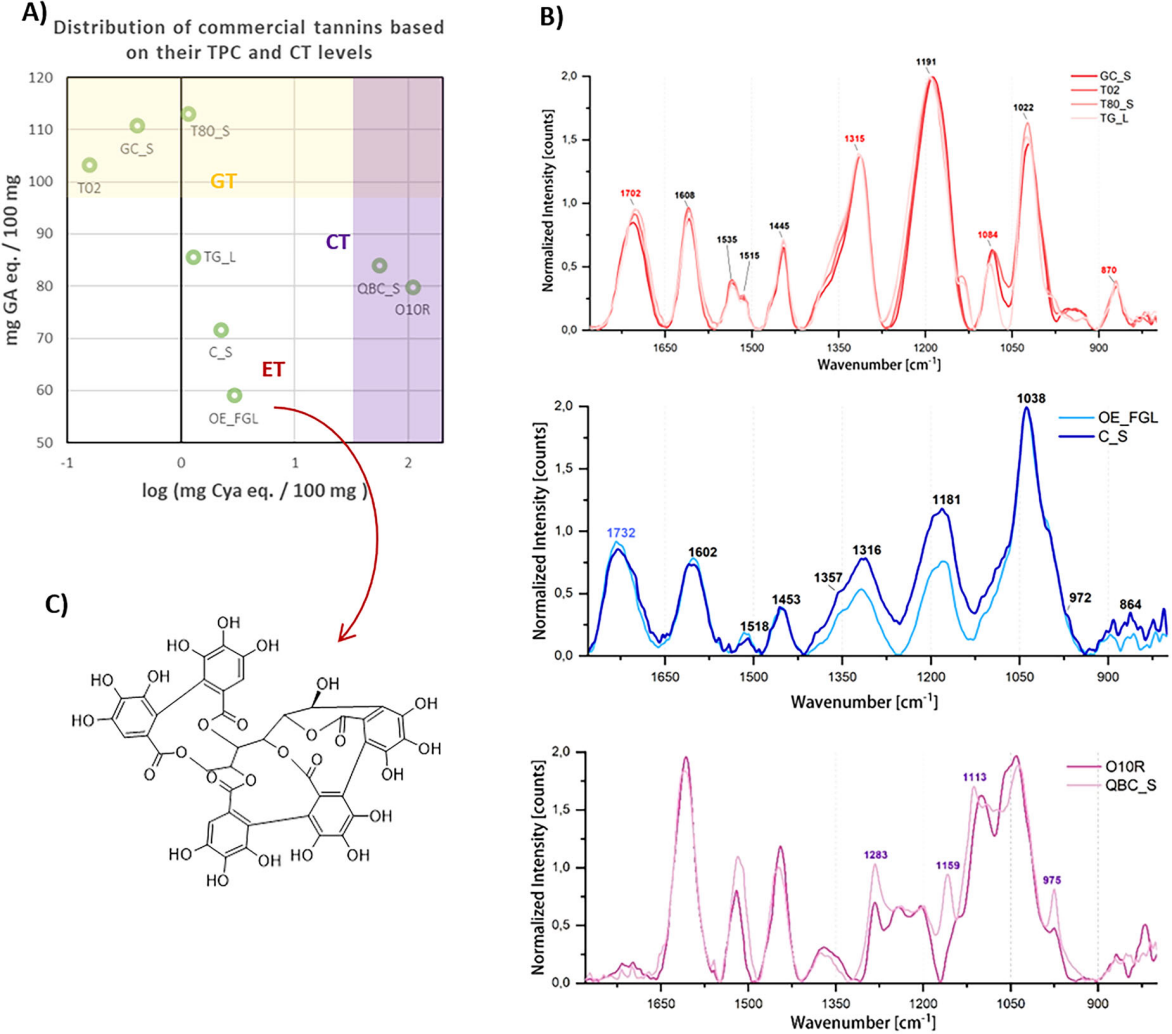
Characterization results of the commercial tannin products used in this study. **A** Distribution of the commercial tannin samples according to their TPC and content of CT, here expressed as log (mg Cya eq./100 mg) for better visualization. **B** ATR-FTIR spectra of the commercial tannin samples. **C** Molecular structure of vescalagin, an ET abundant in oak wood extracts.

The spectrophotometric assays effectively estimated the TPC and CT content; however, these tests do not provide detailed qualitative insights or highly accurate results. The results of the spectrophotometric assays indicate that the commercial tannin samples can be distinguished based on their TPC and CT content, allowing for their classification into distinct groups (Fig. 3A, Table 3). Samples T80_S, GC_S and T02 are characterized by a high TPC (>100 mg GA eq./100 mg of sample) and a negligible content of CT. Samples O10R and QBC_S stand out, as expected, for their high content of CT. Samples TG_L, OE_FGL and C_S can be considered as HT containing a small amount of CT and with a variable TPC ranging from 59.18 to 85.72 mg GA eq./100 mg of sample.

Several studies have demonstrated that the FC method, being based on the reduction of the Folin reagent, is significantly affected by the structural features of the tested polyphenols. In particular, the presence of galloyl groups strongly influences the reducing capacity of the polyphenolic compounds^37–39^. Consequently, the exclusive use of this method may lead to an underestimation of CT and ET^37,39^.

The TPC values obtained through the FC method should therefore be considered as highly indicative and primarily useful for comparative purposes among the analysed samples.

The FT-IR characterization results are in good agreement with the established subdivision of the tannin samples reported above (Fig.3B). For instance, QBC_S and O10R spectra differ from all the other spectra due to the presence of CT markers: signals at about 1283 cm^−1^, 1559 cm^−1^, 1113 cm^−1^ and 975 cm^−1^ are in fact clearly visible in both samples^40–43^. In contrast, the bands at approximately 1700–1735 cm^−1^ and 1315–1320 cm^−1^, which are consistently observed in all other spectra, are characteristic of HT^9,10,41,42,44,45^. Furthermore, the spectra of tannins GC_S, T80, TG_L and T02 exhibit distinct signals at approximately 1085 cm^−1^ and 870 cm^−1^, which are recognized as GT markers^2,41,42^. These markers are absent in OE_FGL and C_S in which ET is expected to be the main constituents. Oak wood extracts are indeed well documented for their high ET content, although CT and GT are also present^46,47^. As an example, Fig. 3C shows one of the most representative ETs identified in oak wood. In addition, water-soluble derivatives of castalagin and vescalagin, such as roburins A–E and grandinin, have been reported in oak heartwood extracts. These compounds contain hexahydroxydiphenoyl (HHDP) esterified units as well as nonahydroxytriphenoyl (NHTP) units, which contribute to their varying metal-binding properties. Furthermore, owing to the distinct structural features of ET, the band above 1700 cm^−1^ attributed to ν (C = O) in esters appears at higher wavenumbers (above 1730 cm^−1^) in OE_FGL and C_S compared to GT.

TG_L, initially grouped with OE_FGL and C_S based on its TPC and CT content, exhibits all characteristic markers of GT. Consequently, the characterization of commercial tannins resulted in the classification of three distinct groups: (i) T80_S, GC_S, T02, and TG_L, which serve as representative GT samples; (ii) QBC_S and O10R, primarily composed of CT; and (iii) C_S and OE_FGL, representative of tannin mixtures containing ET (Fig. 3B).

**Fig. 4:**
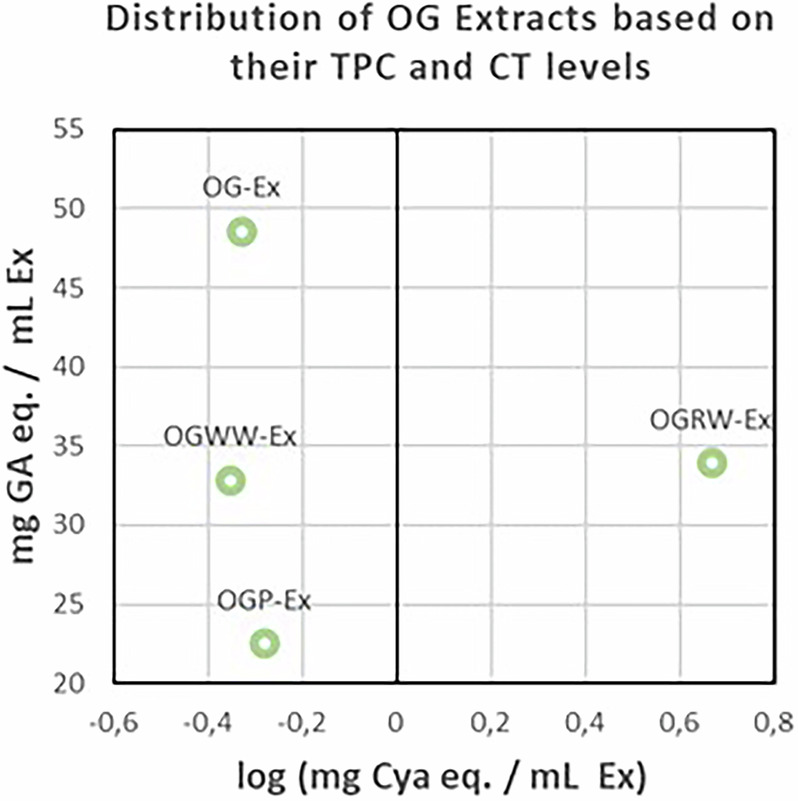
Distribution of OG extracts based on their TPC and CT levels (here expressed as log (mg Cya eq./mL extract) for a better visualization).

For practical reasons, a direct comparison between the TPC and CT content of OG extracts and those of commercial tannin products is not feasible. Nonetheless, the quantification of TPC and CT in OG extracts offers valuable insights into their composition (Fig. 4, Table 4). As expected, OG-Ex, prepared according to the historical “Montpellier” recipe, involving evaporating the extract to one-quarter of its initial volume, exhibits the highest TPC value. The extracts prepared using wine displayed comparable TPC values. Notably, OGRW-Ex, obtained with red wine, also contained a non-negligible amount of CT, as commonly observed in red wines^48^. Finally, OGP-Ex shows a lower TPC, possibly due to the presence of ET and CT. Pomegranate peel is in fact known to be rich in ET, particularly punicalagin, which imparts a vivid yellow coloration to OGP-Ex^49,50^.

**Table 4:** Summary of the results related to OG extracts characterization

Extract	pH	mg of GA eq. per 1 mL	mg of Cya eq. per 1 mL
OG-Ex	3.50 ± 0.02	48.60 ± 1.34	0.47 ± 0.29
OGWW-Ex	3.03 ± 0.08	32.89 ± 0.51	0.44 ± 0.13
OGRW-Ex	3.50 ± 0.01	33.97 ± 1.33	4.64 ± 0.22
OGP-Ex	2.25 ± 0.02	22.59 ± 0.51	0.52 ± 0.17

All reported values result from the average of the triplicate measurements, and the corresponding SD is reported (in the format mean ± SD). See also tables T1.1-T1.3 in the Supporting Materials.

### Characterization of Fe complexes of commercial tannins

**Fig. 5:**
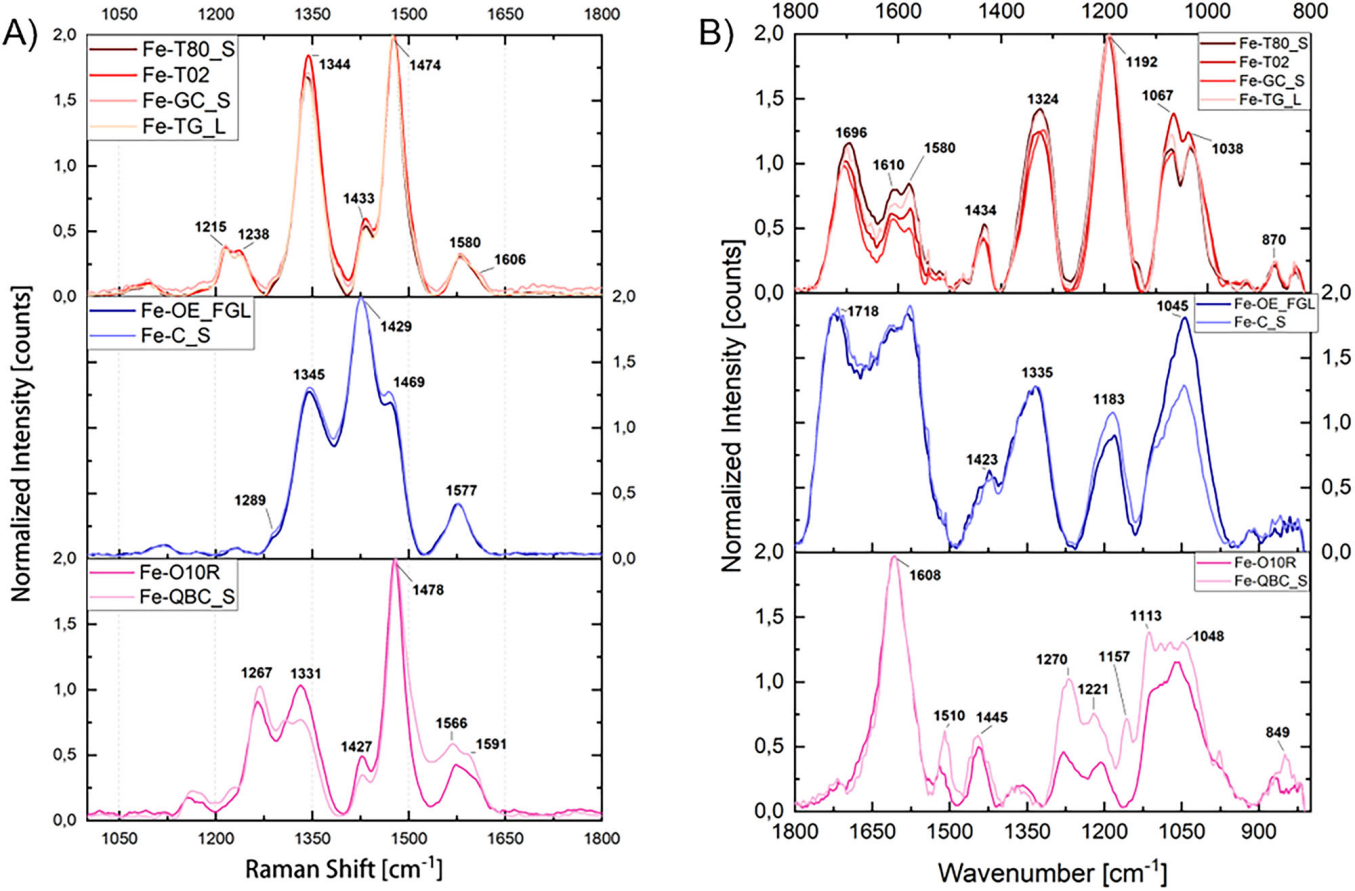
Characterization results of the iron-polyphenolic complexes prepared using commercial tannins. **A** Raman spectra of iron complexes from commercial tannins. Each spectrum represents the average of five spectra acquired per pellet, with pellets prepared in triplicate. **B** ATR-FT-IR spectra of iron complexes from commercial tannins. Each spectrum shown is an average of 5–6 selected spectra per pellet, chosen from a total of 1024 spectra collected using the FPA detector. Pellets were prepared in triplicate.

The Raman spectroscopy characterization of Fe-polyphenolic complexes prepared using commercial tannins (Table  1) reveals significant spectral differences that align with the previously discussed tannin characterization (Fig. 5A). The spectra for the iron complexes of GC_S, T80_S, G_L, and T02 exhibit typical Fe-GT complex features, as reported in the literature: two strong peaks around 1470–1480 cm^−1^ and 1330–1350 cm^−1^, corresponding to aromatic ring stretching vibrations and C-O stretching of the phenolic moieties, a shoulder at about 1430 cm^−1^ associated with COO vibrations, and two medium-intensity broader bands at approximately 1220-1240 cm^−1^ (often as a doublet) and 1580 cm^−1^ ^3,7^. Recent studies have demonstrated that the band primarily associated with ν(C-O) vibrations is particularly sensitive to the structural characteristics of the polyphenolic ligand^7,9^. In all analysed iron-commercial tannins complexes, this band appears at approximately 1345 cm^−1^, a value comparable to that observed for iron complexes of tannic acid, suggesting that the GT involved as ligands are high-molecular-weight (MW) polyphenols^7,9^. Conversely, in OG extracts, where the predominant GT species have a lower MW, the ν(C-O) band typically shifts to lower wavenumbers, around 1330 cm^−1^ ^2,7,30^. A further shift to even lower wavenumbers is observed when GA serves as the primary ligand, around 1315 cm^−1^, as observed in the so-called “*gallic acid inks*”^7,32,51,52^.

In contrast, the spectra of Fe-O10R and Fe-QBC_S exhibit notable differences: the 1340 cm^−1^ signal is shifted and its intensity is significantly reduced. Moreover, the doublet around 1220–1240 cm^−1^ observed in Fe-GT complexes merges into a single medium-intensity band, centered at approximately 1260 cm^−1^ in these samples. This band has recently been assigned to ν (C-O) vibrations coupled with ring stretching vibrations and δ(C-H)^10^, but further studies are necessary to refine the assignments of the peaks in the range 1200–1250 cm^−1^. Nevertheless, similar spectra have recently been reported by *Derkacheva* et al. for IGI prepared according to historical Russian recipes, in which alder bark decoction serves as a source of polyphenolic ligands^53^. The presence of two peaks of comparable intensity, centered around 1260 cm^−1^ and 1330 cm^−1^, can thus be considered as a characteristic marker of Fe-CT.

The spectra of iron complexes with OE_FGL and C_S also show distinct differences in the main peaks, supporting the tannin characterization results. Notably, the main signal of aromatic stretching vibrations around 1470 cm^–1^ appears as a shoulder to the strong 1430 cm^−1^ band, which was only a minor shoulder in the Fe-GT spectra. The results reported by *Henrik-Klemens* et al. indicate that even in iron complexes of oak tannins from real archaeological samples, the 1430 cm^−1^ peak exhibits greater intensity than the 1340 cm^−1^ peak^54^. Comparable results were also obtained for iron complexes derived from natural extracts of various ET-rich matrices, including chestnuts, valonia, and myrobalan^31^. The intensity ratio between the peaks at approximately 1430 cm^−1^ and 1340 cm^−1^ serves therefore as a reliable marker for Fe-ET.

The IR results support this subdivision as well (Fig. 5B). The spectral profiles of Fe-GT, Fe-ET, and Fe-CT exhibit distinct differences, allowing for clear differentiation among them. As reported in the literature, the metal complexation of large polyphenols induces only minor changes in the IR spectral profile within the 1800–400 cm^−1^ range^7^. The most affected bands are those primarily associated with ν(C–O) vibrations or influenced by them, leading to slight modifications in the spectral profile. Nevertheless, the characteristic HT, GT, and CT spectral markers previously identified (Table 5) remain clearly distinguishable in the iron-complexed forms of tannins (see Figure F.1 in the Supporting Materials).

**Table 5:** FT-IR bands in tannins. Band assignments were determined based on literature data^9,40–42,44^

Band position (cm^−1^)	Assignment	Band occurrence
1675–1735	ν(C = O) esters	HT marker
1605–1625	Aromatic stretching vibrations	Common to all tannins
1510–1535	Skeletal aromatic ring vibrations	
1440–1450	Aromatic stretching vibrations	
1315–1345	ν(C-OC) + δ(OH)	HT marker
1280–1285	ν(C-O) pyran ring	CT marker
1185–1215	ν(C-O) phenols	Common to all tannins
1150–1160	ν(C-O-C) cyclic ether	CT markers
1110–1120		
1080–1085	ν(C-O-C) phenolic esters	GT marker
1020–1045	ν(C-O) phenols / δ(=C-H)^a^	Common to all tannins
975–980	n.a.	CT marker
870–875	Aromatic δ(C-H) + δ(OH)	GT maker
835–845	Aromatic δ(C-H)	CT marker

^a^In this case, the assignment is not consistently reported in the literature; however, this band is widely recognized as a characteristic feature of natural tannins.

**Fig. 6:**
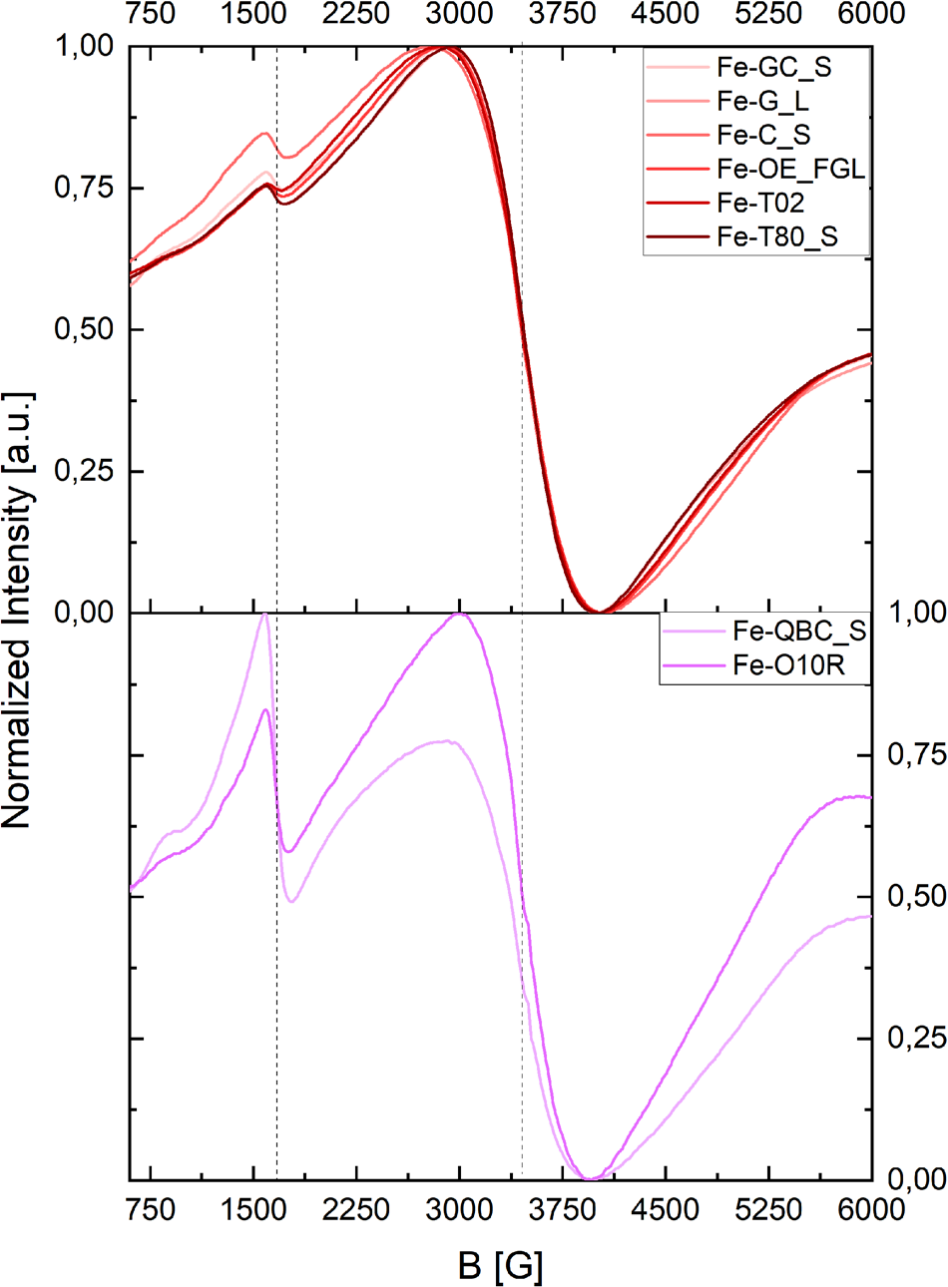
CW-EPR spectra of iron complexes of commercial tannins. The spectra shown have been initially normalized by the sample weights and just for a better visualization, a further normalization between 0 and 1 has been performed (see also Fig. F.2 in the Supporting Materials). The two vertical dotted lines highlight the inflection points of the two main signals at g ≈ 2 and g ≈ 4.3.

The results of the EPR characterization are presented in Fig. 6 (see also Supporting Materials Figure F.2). All reported spectra are dominated by the Fe³⁺ high-spin (S = 5/2) signal at g ≈ 2 (~3500 G). This signal is typically associated with a pseudo-octahedral coordination geometry of the iron center, which represents the most stable and common configuration for Fe³⁺ (*d*⁵ transition metal)^7^. Notably, all spectra also exhibit a signal in the region of g ≈ 4.3, which has been attributed to a tetrahedral coordination environment with a strong rhombic distortion^7,55–57^. The comparison shown in Fig. 6 indicates that the qualitative intensity ratio between these two main signals allows the discrimination between complexes of HT and CT, while no significant differences were observed between the spectra of Fe-GT and Fe-ET. This observation aligns with previous studies on logwood inks, where the iron-coordinating polyphenols, although distinct from tannins, share structural similarities with CT monomers^55^. The qualitative proportion of the two signals can therefore be used as a marker of Fe-CT.

### Characterization of Fe complexes prepared from OG extracts

The identification of those spectral markers which could enable a discrimination among Fe-polyphenolic complexes (Tab. 2) is for sure challenging in OG extracts in which the GT markers, and consequently also the HT markers, are expected.

**Fig. 7:**
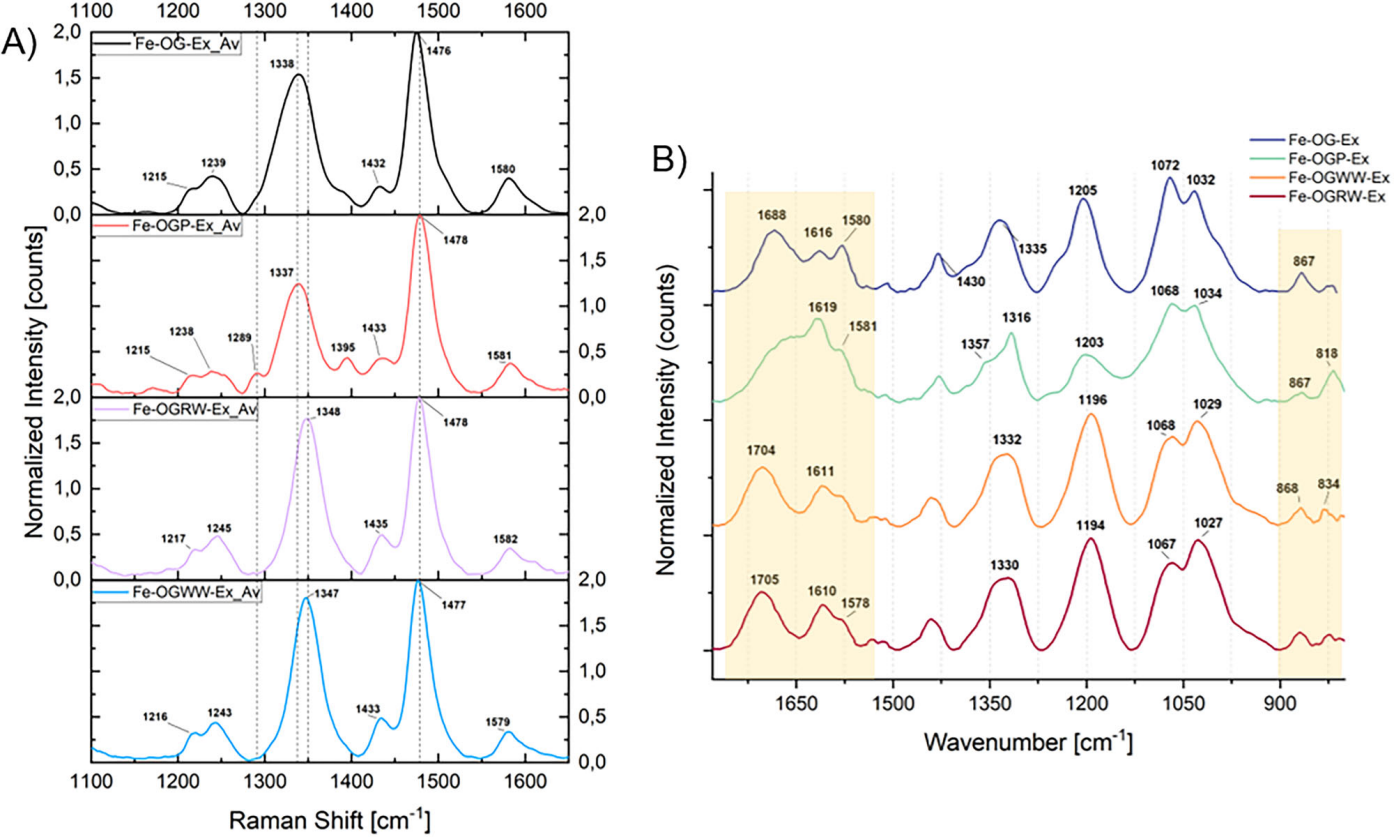
Characterization results of the iron-polyphenolic complexes obtained starting from OG extracts following historical recipes. **A** Raman spectra of the iron complexes prepared with the different OG extracts. Each spectrum represents the average of 9 measurements (3 spectra per pellet, 3 pellets per formulation). **B** ATR-FTIR spectra of the iron complexes prepared with the different OG extracts, averaged from 15 measurements per sample set (5 spectra per pellet, 3 pellets per formulation). Minimal spectral variability was observed within each subset (see Fig. F. 3 in the Supporting Materials).

The comparison of the Raman spectra reveals that the band primarily associated with the ν(C–O) stretching (generally lying in the range 1330 – 1350 cm^-1^) appears at higher wavenumbers (by ~10 cm^−1^) in the complexes prepared with extracts containing wine, compared to the other two complexes (Fig. 7A). Moreover, in the IR spectra, the spectral profiles of the wine-based complexes, which are nearly identical, exhibit a slight shift (~20 cm^−1^) of the ester ν(C = O) band towards higher wavenumbers compared to the other samples. These shifts may be attributed to the presence of higher molecular weight tannins in the polyphenolic fraction of the wine extracts (Fig. 7B; see also Fig. F2 in Supporting Materials). An additional Raman band at ~1289 cm^−1^ is observed in the complexes prepared with the OGP-Ex, which is not clearly discernible in the spectra of the other samples. A comparable feature was previously noted as a weak shoulder in Fe-ET complexes prepared with commercial tannins (Fig. 3B). Furthermore, consistent with the reference spectra of iron-commercial tannin complexes, the IR spectrum of Fe-OGP-Ex displays a broad absorption envelope in the 1730–1560 cm^−1^ range, from which only the band at ~1610–1620 cm^−1^ can be clearly resolved. The IR region between 900 and 800 cm^−1^ is consistent with previously established markers: the complex prepared with the general extract displays a medium-weak band at ~870 cm^−1^, while the wine-based complexes also feature an additional band at ~834 cm^−1^.

Overall, the vibrational spectroscopic data suggest that when tannins other than GT are present only in minor amounts in the natural extracts, the identification of previously proposed markers (Table 5) may not be feasible. Although the position of the Raman ν(C–O) band varies across formulations, this shift alone cannot reliably distinguish between inks due to its susceptibility to multiple factors^7^. Similarly, IR spectroscopy provides useful insights, even though the identification of definitive markers remains challenging. The spectral regions 1780–1560 cm^−1^ and 900–800 cm^−1^ appear to be the most informative in this context.

**Fig. 8:**
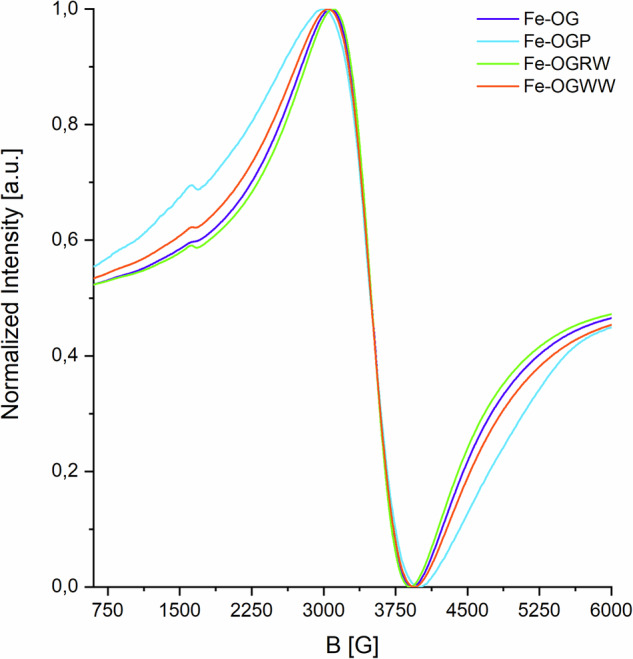
CW-EPR spectra of Fe-polyphenolic complexes prepared using OG extracts. The spectra shown undergone the same type of double normalization reported in Fig. 6.

All spectra acquired by EPR spectroscopy are characterized by the dominant signal at g ≈ 2 accompanied by only a faint signal attributable to a tetrahedral coordination geometry (g ≈ 4.3, Fig. 8; see also F.4 in the Supporting Materials). These findings support the observation that, in the sample in which only a small fraction of iron is complexed with polyphenolic compounds other than GT, it remains challenging to identify spectroscopic markers that allow for a reliable differentiation without invasive approaches, even with a sensitive technique such as CW-EPR.

It should be noticed that the results of these characterization are dependent on the sample preparation methodology. In the applied protocol, once formed and oxidized for 10–15 days, the complexes have been isolated via centrifugation-washing cycles. As a result, the recovered solid precipitates contain only those compounds that had formed solid complexes with Fe, of sufficient particle size, by the time of centrifugation. Other extract components that did not participate in complexation under these conditions were removed during this step. In IGIs, however, these un-complexed compounds would likely be present, potentially enhancing the visibility of the spectral markers previously identified.

From a chemical perspective, these findings suggest that in systems where multiple polyphenolic ligands compete to form iron–polyphenol complexes, the formation of iron–galloyl complexes is favoured. This preference is likely driven by the higher abundance of galloyl groups compared to catechol moieties.

### Characterization of 15th century manuscript fragments

**Fig. 9:**
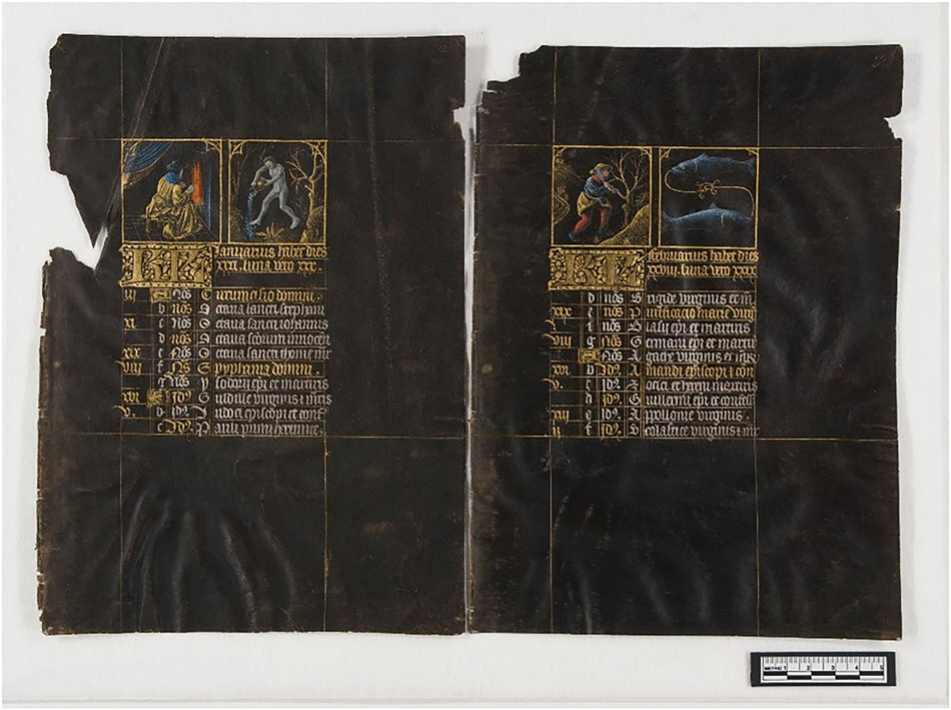
Folios 2r and 3r of the Codex 1856 (Vienna, ÖNB) depict the calendar pages for January and February. Copyright ÖNB.

Three small fragments recovered during prior conservation treatments from the 1856 Codex property of the Austrian National Library(ÖNB), a luxurious prayer book also known as the “Black Hours”, were analysed (see also figure F.5 in the Supporting Materials). This manuscript is distinguished by its pages, entirely coated with IGI, and text as well as illuminations rendered in silver- and gold-based inks, contributing to its striking visual impact (Fig. 9). Due to its artistic and historical significance, as well as the severe deterioration it has undergone, previous analytical investigations have been conducted to elucidate the chemical composition of its inks and pigments and to assess the condition of the parchment^35^. In this previous analytical campaign, both XRF and preliminary EPR measurements highlight the presence of copper in a relatively high amount in the IGI used to colour the parchment pages^35^. Within this framework, the present study enabled a more detailed re-examination of the fragments using the previously described multi-analytical approach (Raman, ATR-FTIR, and CW-EPR), with the aim of gaining insights into the materials used in the manuscript’s production, specifically the polyphenolic component of the IGI extensively applied to the folios. This information may support the preparation of appropriate mock-ups for testing potential treatments and storage materials. Additionally, understanding the materials employed may provide archaeometric insights about the art technology behind this object.

**Fig. 10:**
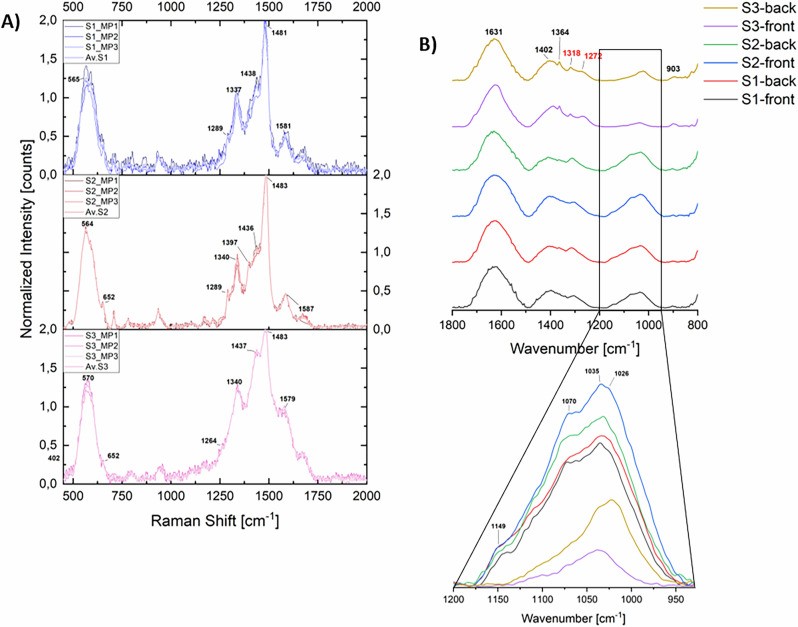
Raman and FTIR spectra acquired on the 1856 Codex’ fragments. **A** Raman spectra acquired on the historical manuscript fragments. **B** ATR-IR spectra of the three historical manuscript fragments. S1 = sample 1, S2 = sample 2, S3 = sample 3. See also F. 2 in the Supporting Materials.

The Raman spectra acquired are presented in Fig. 10A. The intensity of the signal associated with ν(C-O) vibrations of phenolic moieties, observed in all spectra at ~1340 cm^−1^, is significantly lower compared to the band at ~1480 cm^−1^, which is primarily attributed to aromatic vibrations. The intensity ratio between these two bands, along with the pronounced signal at ~560–570 cm^−1^, are characteristic features commonly observed in ancient manuscripts, where oxidation processes lead to a decrease in the 1340 cm^−1^ signal. The band at ~1430 cm^−1^ is particularly intense across all spectra. Such high intensities have been previously reported in historical samples^3,31,33^, and may be associated with hydrolysis processes, which enhance the intensity of ν(COO) vibrations. Overall, the Raman analysis of these samples highlights the extensive oxidation undergone by the IGI.

The IR spectra shown in Fig. 10B are, as expected, strongly influenced by vibrational bands characteristic of parchment collagen^⁵⁷^. These include, in particular, the intense and broad amide ν(C = O) band centered around 1630 cm^−1^ and a series of bands in the 1100–1000 cm^−1^ region, which largely obscure the signals associated with the ink^58,59^. Nevertheless, within the 1200–950 cm^−1^ range, one of the characteristic GT bands remains observable at approximately 1070 cm^−1^. Additionally, a weak shoulder at ~1150 cm^−1^, more evident in samples S1 and S2, may be attributed to a CT marker, specifically the ν(C–O–C) cyclic ether vibration (see also Table 5).

**Fig. 11:**
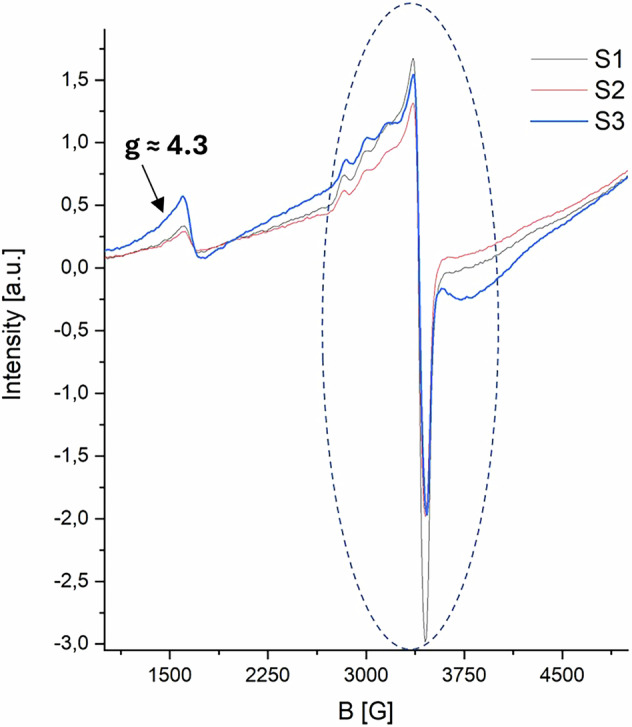
CW-EPR spectra of the three manuscript fragments. Circled in dotted line, the signals related to Cu2+ coordinated cations.

The CW-EPR spectra, consistent with previous findings^35^, are dominated by intense signals attributed to copper ions (Fig. 11)^55,60^. These signals fully obscure the g ≈ 2 iron-related region; however, the g ≈ 4.3 signal remains detectable in all three spectra and may correspond to rhombically distorted iron species. Based on both the profile and intensity of these signals, particularly in sample S3, the presence of CT is suggested, as it likely induces a strong rhombic distortion in the paramagnetic iron centres of the complexes (see also Fig. 5).

Considering all the results reported and discussed here the possible presence of Fe-CT complexes could be suggested, together with Fe-GT and Cu-GT. However, in the absence of historical or philological information, such as potential recipes or materials used in the manuscript’s production, any hypothesis remains speculative unless supported by more detailed analyses, potentially requiring invasive and destructive methods such as MS based techniques. For example, it has been demonstrated that the polyphenolic composition of OG and consequently that of OG extracts, varies depending on the provenance and maturity of the galls^61–64^. Notably, previous studies have identified the presence of CT in Italian OG^56^. For this reason, MS-based techniques are recommended to obtain more detailed insights for identifying specific tannin sources and additives.

## Discussion

Throughout the study of iron complexes of different commercial tannins and laboratory-prepared OG extracts, it has been possible to highlight the potentialities as well as the limitations of Raman spectroscopy, ATR-FTIR and CW-EPR in the study of the polyphenolic components of IGI. The characterization of complexes prepared using commercial tannins allowed for the identification of Fe-GT, Fe-CT, and Fe-ET spectral markers across all three techniques, in good agreement with the existing literature. It was shown that Raman and IR techniques are capable of discriminating among these three types of iron–tannin complexes, whereas EPR can distinguish only between Fe-HT and Fe-CT, due to the pronounced rhombic distortion induced by the structure of CT.

However, the characterization of the iron complexes of OG extracts highlighted the challenges in the identification of the specific Fe-tannins spectral markers in complexes in which tannins other than GT are present just in small quantities and always in the presence of higher quantities of GT. The limitations of these techniques have been highlighted and should be considered as guidelines in future studies aimed at investigating the organic components of IGI from this perspective. None of the three non-invasive techniques yielded sufficiently robust data to allow reliable differentiation: slight shifts of the Raman band around 1330 cm^−1^ and variations in ATR-FTIR profiles between 1780–1560 and 900–800 cm^−1^ were observed, but the limited extent of these differences and the variability of the data preclude confident discrimination of IGI^7^. It should be noted that, in general, the variability of data acquired from IGI-containing manuscripts is significantly influenced by multiple factors, including the precision of the instrumental technique, the typology of the ink support, surface heterogeneity, and ongoing degradation processes affecting the manuscript. Therefore, the variability observed in the un-aged ink pellets used in this study should be considered substantially lower than that typically observed in historical manuscripts. Nevertheless, the analysis of fragments from an ancient manuscript confirmed that all three techniques investigated can provide relevant insights into the polyphenolic components of IGI, even in degraded samples. Nonetheless, without the support of additional and more robust evidence, attributing the presence of specific iron–tannin complexes and, by extension, specific additives used in historical ink recipes remains highly speculative.

Such information is critical from both archaeometric and conservation standpoints, as it has been shown that the effectiveness of conservation treatments, such as the removal of excess iron, can be influenced by the structural characteristics of the polyphenolic ligands present in the ink^65^. Therefore, well-supported and corroborated evidence should be considered essential in IGI-related studies to ensure reliable interpretations.

## Supplementary information

Supplementary materials

## Data Availability

All data generated or analysed during this study are included in this published article and its supplementary information file.

## References

[1] Caterino, S., Pajer, N. & Crestini, C. Iron-galls inks: preparation, structure and characterisation. *Microchem. J.* **185**, 108258 (2023).

[2] Díaz Hidalgo, R. J. et al. New insights into iron-gall inks through the use of historically accurate reconstructions. *Herit. Sci.* **6**, 63 (2018).

[3] Ponce, A. et al. Elucidation of the Fe(III) gallate structure in historical iron gall ink. *Anal. Chem.* **88**, 5152–5158 (2016). 27058399 10.1021/acs.analchem.6b00088

[4] Díaz Hidalgo, R. J. et al. The making of black inks in an Arabic treatise by Al-Qalalūsī dated from the 13th c.: reproduction and characterisation of iron-gall ink recipes. *Herit. Sci.* **11**, 7 (2023).

[5] Vassiou, E., Lazidou, D., Kampasakali, E., Pavlidou, E. & Stratis, J. Iron gall ink from historical recipes on organic substrates and their study before and after accelerated ageing with Μ-RAMAN spectroscopy and SEM-EDS. *J. Cult. Herit.* **66**, 584–592 (2024).

[6] Perron, N. R. & Brumaghim, J. L. A review of the antioxidant mechanisms of polyphenol compounds related to iron binding. *Cell Biochem. Biophys.* **53**, 75–100 (2009). 19184542 10.1007/s12013-009-9043-x

[7] Caterino, S. et al. A systematic multianalytical approach in the study of iron–polyphenolic complexes in iron-gall inks: exploring the potentialities of Raman and electron paramagnetic resonance. *Inorg. Chem*. acs.inorgchem.4c04232. 10.1021/acs.inorgchem.4c04232 (2025). 10.1021/acs.inorgchem.4c04232 40029985

[8] Malacaria, L. et al. A review on coordination properties of Al(III) and Fe(III) toward natural antioxidant molecules: experimental and theoretical insights. *Molecules* **26**, 2603 (2021). 33946938 10.3390/molecules26092603 PMC8124610

[9] Espina, A., Cañamares, M. V., Jurašeková, Z. & Sanchez-Cortes, S. Analysis of iron complexes of tannic acid and other related polyphenols as revealed by spectroscopic techniques: implications in the identification and characterization of iron gall inks in historical manuscripts. *ACS Omega* **7**, 27937–27949 (2022). 35990485 10.1021/acsomega.2c01679 PMC9386834

[10] Espina, A., Sanchez-Cortes, S. & Jurašeková, Z. Vibrational study (Raman, SERS, and IR) of plant gallnut polyphenols related to the fabrication of iron gall inks. *Molecules* **27**, 279 (2022). 35011511 10.3390/molecules27010279 PMC8746386

[11] Preparation of Black Ink. Harper’s Bazar. pp. 113–114. (Hearst Corporation, 1880).

[12] Contreras Zamorano, G. M. Evolución de La Composición de Las Tintas Ferrogálicas a Través de Las Fuentes Documentales de Los Siglos XIII al XIX. Meridies, 34–67. 10.21071/meridies.vi13.14250 (2022).

[13] Retko, K., Legan, L., Kosel, J. & Ropret, P. Identification of iron gall inks, logwood inks, and their mixtures using Raman spectroscopy, supplemented by reflection and transmission infrared spectroscopy. *Herit. Sci.* **12**, 212 (2024).

[14] Ferretti, A., Sabatini, F. & Degano, I. Linking historical recipes and ageing mechanisms: the issue of 19th century iron gall inks. *J. Cult. Herit.* **67**, 111–120 (2024).

[15] Le Bourvellec, C., Renard, C. M. G. C. Interactions between polyphenols and macromolecules: effect of tannin structure. In Encyclopedia of Food Chemistry; pp 515–521. 10.1016/B978-0-08-100596-5.21486-8 (Elsevier, 2019).

[16] Le Bourvellec, C. & Renard, C. M. G. C. Interactions between polyphenols and macromolecules: quantification methods and mechanisms. *Crit. Rev. Food Sci. Nutr.* **52**, 213–248 (2012). 22214442 10.1080/10408398.2010.499808

[17] Brglez Mojzer, E., Knez Hrnčič, M., Škerget, M., Knez, Ž & Bren, U. Polyphenols: extraction methods, antioxidative action, bioavailability and anticarcinogenic effects. *Molecules* **21**, 901 (2016). 27409600 10.3390/molecules21070901 PMC6273793

[18] Ajila, C. M. et al. Extraction and analysis of polyphenols: recent trends. *Crit. Rev. Biotechnol.* **31**, 227–249 (2011). 21073258 10.3109/07388551.2010.513677

[19] Khanbabaee, K. & Ree, T. Tannins: classification and definition. *Nat. Prod. Rep.* **18**, 641–649 (2001). 11820762 10.1039/b101061l

[20] Okuda, T., Yoshida, T. & Hatano, T. Classification of oligomeric hydrolysable tannins and specificity of their occurrence in plants. *Phytochemistry* **32**, 507–521 (1993).

[21] Serrano, J., Puupponen-Pimiä, R., Dauer, A., Aura, A.-M. & Saura-Calixto, F. Tannins: current knowledge of food sources, intake, bioavailability and biological effects. *Mol. Nutr. Food Res.* **53**, S310–S329 (2009). 19437486 10.1002/mnfr.200900039

[22] Wall-Medrano, A. Taninos hidrolizables; bioquímica, aspectos nutricionales y analíticos y. Nutricion hospitalaria, 55–66. 10.3305/nh.2015.31.1.7699 (2015). 10.3305/nh.2015.31.1.7699 25561098

[23] Haslam, E. & Cai, Y. Plant polyphenols (vegetable tannins): gallic acid metabolism. *Nat. Prod. Rep.* **11**, 41 (1994). 15206456 10.1039/np9941100041

[24] Xie, D.-Y. & Dixon, R. A. Proanthocyanidin biosynthesis—still more questions than answers?. *Phytochemistry* **66**, 2127–2144 (2005). 16153412 10.1016/j.phytochem.2005.01.008

[25] Ferreira, D.; Bekker, R. Oligomeric proanthocyanidins: naturally occurring o-heterocycles. *Nat. Prod. Rep*. **23** (1996). 10.1039/a705728h 10821113

[26] Lopes, G. et al. Can phlorotannins purified extracts constitute a novel pharmacological alternative for microbial infections with associated inflammatory conditions?. *PLoS ONE* **7**, e31145 (2012). 22319609 10.1371/journal.pone.0031145 PMC3271118

[27] Duh, J., Krstić, D., Desnica, V. & Fazinić, S. Non-Destructive Study of Iron Gall Inks in Manuscripts. *Nuclear Instruments and Methods in Physics Research Section B: Beam Interactions with Materials and Atoms.* 417, 96–99 (2018)

[28] Prochet, B. et al. Non-destructive characterisation of inks by spectroscopy and surface analyses. In 2020 IEEE International Conference on Environment and Electrical Engineering and 2020 IEEE Industrial and Commercial Power Systems Europe (EEEIC / I&CPS Europe); IEEE: Madrid, Spain; pp 1–5. 10.1109/EEEIC/ICPSEurope49358.2020.9160578 (2020).

[29] Goltz, D. M. A review of instrumental approaches for studying historical inks. *Anal. Lett.* **45**, 314–329 (2012).

[30] Teixeira, N., Nabais, P., de Freitas, V., Lopes, J. A. & Melo, M. J. In-depth phenolic characterization of iron gall inks by deconstructing representative Iberian recipes. *Sci. Rep.* **11**, 8811 (2021). 33893347 10.1038/s41598-021-87969-3 PMC8065154

[31] Bicchieri, M., Monti, M., Piantanida, G. & Sodo, A. Non-destructive spectroscopic investigation on historic Yemenite scriptorial fragments: evidence of different degradation and recipes for iron tannic inks. *Anal. Bioanal. Chem.* **405**, 2713–2721 (2013). 23307133 10.1007/s00216-012-6681-4

[32] Ferretti, A., Sabatini, F. & Degano, I. A model iron gall ink: an in-depth study of ageing processes involving gallic acid. *Molecules* **27**, 8603 (2022). 36500696 10.3390/molecules27238603 PMC9735674

[33] Piantanida, G., Menart, E., Bicchieri, M. & Strlič, M. Classification of iron-based inks by means of micro-Raman spectroscopy and multivariate data analysis: classification of iron-based inks by means of micro-Raman spectroscopy and multivariate data analysis. *J. Raman Spectrosc.* **44**, 1299–1305 (2013).

[34] Corregidor, V., Viegas, R., Ferreira, L. M. & Alves, L. C. Study of iron gall inks, ingredients and paper composition using non-destructive techniques. *Heritage* **2**, 2691–2703 (2019).

[35] Hofmann, C. et al. The black hours: material and conservation study, part 1. *J. Paper Conserv*. 1–18. 10.1080/18680860.2024.2420274 (2024).

[36] Santoso, S. P. et al. Unlocking the potential of gallic acid-based metal phenolic networks for innovative adsorbent design. *Molecules* **30**, 1218 (2025). 40141997 10.3390/molecules30061218 PMC11945622

[37] Pérez, M., Dominguez-López, I. & Lamuela-Raventós, R. M. The chemistry behind the Folin–Ciocalteu method for the estimation of (poly)phenol content in food: total phenolic intake in a Mediterranean dietary pattern. *J. Agric. Food Chem.* **71**, 17543–17553 (2023). 37948650 10.1021/acs.jafc.3c04022 PMC10682990

[38] Martins, G. R., Monteiro, A. F., do Amaral, F. R. L. & da Silva, A. S. A validated folin-ciocalteu method for total phenolics quantification of condensed Tannin-Rich Açaí (Euterpe Oleracea Mart.) seeds extract. *J. Food Sci. Technol.* **58**, 4693–4702 (2021). 34629533 10.1007/s13197-020-04959-5 PMC8479047

[39] Platzer, M., Kiese, S., Herfellner, T., Schweiggert-Weisz, U. & Eisner, P. How does the phenol structure influence the results of the Folin-Ciocalteu assay. *Antioxidants* **10**, 811 (2021). 34065207 10.3390/antiox10050811 PMC8160659

[40] Falcão, L. & Araújo, M. E. M. Tannins characterization in historic leathers by complementary analytical techniques ATR-FTIR, UV-Vis and chemical tests. *J. Cult. Herit.* **14**, 499–508 (2013).

[41] Falcão, L. & Araújo, M. Vegetable tannins used in the manufacture of historic leathers. *Molecules* **23**, 1081 (2018). 29751585 10.3390/molecules23051081 PMC6099987

[42] Koochakzaei, A. & Sabaghian, M. Tannin characterization and sourcing in historical leathers through FTIR spectroscopy and PCA analysis. *Collagen Leather* **5**, 21 (2023).

[43] Arshad, M., Beg, A. & Siddiqui, Z. A. Infrared spectroscopic investigation of tannins. *Angew. Makromol. Chem.* **7**, 67–78 (1969).

[44] Çakar, S., Güy, N., Özacar, M. & Fındık, F. Investigation of vegetable tannins and their iron complex dyes for dye sensitized solar cell applications. *Electrochim. Acta* **209**, 407–422 (2016).

[45] Çakar, S. & Özacar, M. The PH dependent tannic acid and fe-tannic acid complex dye for dye sensitized solar cell applications. *J. Photochem. Photobiol. A Chem.* **371**, 282–291 (2019).

[46] García-Estévez, I., Escribano-Bailón, M. T., Rivas-Gonzalo, J. C. & Alcalde-Eon, C. Development of a fractionation method for the detection and identification of oak ellagitannins in red wines. *Anal. Chim. Acta* **660**, 171–176 (2010). 20103159 10.1016/j.aca.2009.10.020

[47] Puech, J.-L., Feuillat, F. & Mosedale, J. R. The tannins of oak heartwood: structure, properties, and their influence on wine flavor. *Am. J. Enol. Vitic.* **50**, 469–478 (1999).

[48] Watrelot, A. A. Tannin content in Vitis species red wines quantified using three analytical methods. *Molecules* **26**, 4923 (2021). 34443511 10.3390/molecules26164923 PMC8400854

[49] Oudane, B. Isolation, characterization, antioxidant activity, and protein-precipitating capacity of the hydrolyzable tannin punicalagin from pomegranate yellow peel (*Punica Granatum*). *J. Mol. Struct*. **1156**, 390-396 (2018).

[50] Saad, H. et al. Characterization of pomegranate peels tannin extractives. *Ind. Crops Prod.* **40**, 239–246 (2012).

[51] Lee, A. S., Mahon, P. J. & Creagh, D. C. Raman analysis of iron gall inks on parchment. *Vib. Spectrosc* **41**, 170–175 (2006).

[52] Lehner Sigmund. The manufacture of ink. (Collins Printing House, 1892).

[53] Derkacheva, O. Y., Lotsmanova, E. M. & Bystrova, E. S. Micro-Raman spectroscopy of replicated iron ink. *Nanotechnol. Russ.* **19**, 509–516 (2024).

[54] Henrik-Klemens, Å., Bengtsson, F., Björdal, C. G. Raman spectroscopic investigation of iron-tannin precipitates in waterlogged archaeological oak. *Studies Conserv*. 1–11. 10.1080/00393630.2020.1864895 (2021).

[55] Bronzato, M., Zoleo, A., Biondi, B. & Centeno, S. A. An insight into the metal coordination and spectroscopic properties of artistic Fe and Fe/Cu Logwood Inks. *Spectrochim. Acta Part A Mol. Biomol. Spectrosc.* **153**, 522–529 (2016). 10.1016/j.saa.2015.08.042 26414555

[56] Caterino, S. Elucidation of the structure of iron-gall inks by an innovative multi-analytical approach: unexpected new insights into the chemistry of iron polyphenolic complexes revealed by NMR and EPR spectroscopy, Ca’ Foscari, Venezia. https://hdl.handle.net/20.500.14247/6984 (2022).

[57] Ciglanská, M., Jančovičová, V., Havlínová, B., Machatová, Z. & Brezová, V. The influence of pollutants on accelerated ageing of parchment with iron gall inks. *J. Cultural Herit.* **15**, 373–381 (2014).

[58] Boyatzis, S. C., Velivasaki, G. & Malea, E. A study of the deterioration of aged parchment marked with laboratory iron gall inks using FTIR-ATR spectroscopy and micro hot table. *Herit. Sci.* **4**, 13 (2016).

[59] Malea, E. et al. The complementary use of Raman, ATR-FTIR spectroscopy, and chemometrics for investigating the deterioration of artificially aged parchment. *J. Raman Spectrosc.* **55**, 1266–1280 (2024).

[60] Punis, R. & Zoleo, A. Cu(II)-binder complexes in azurite and malachite pictorial mixtures: an EPR study. *Microchem. J.* **200**, 110303 (2024).

[61] Hartley, S. E. The chemical composition of plant galls: are levels of nutrients and secondary compounds controlled by the gall-former?. *Oecologia* **113**, 492–501 (1998). 28308028 10.1007/s004420050401

[62] Chen, H. et al. Molecular mechanisms of tannin accumulation in Rhus galls and genes involved in plant-insect interactions. *Sci. Rep.* **8**, 9841 (2018). 29959354 10.1038/s41598-018-28153-y PMC6026138

[63] Melone, F., Saladino, R., Lange, H. & Crestini, C. Tannin structural elucidation and quantitative ^31^ P NMR analysis. 2. Hydrolyzable tannins and proanthocyanidins. *J. Agric. Food Chem.* **61**, 9316–9324 (2013). 23998855 10.1021/jf401664a

[64] Banc, R., Rusu, M. E., Filip, L. & Popa, D.-S. Phytochemical profiling and biological activities of Quercus Sp. Galls (Oak Galls): a systematic review of studies published in the last 5 years. *Plants* **12**, 3873 (2023). 38005770 10.3390/plants12223873 PMC10674842

[65] Costa, A. et al. Archaeometric investigations on naturally and thermally-aged iron-gall inks using different tannin sources. *Open Chem.* **11**, 1729–1739 (2013).

