# Supplementary material for: Non-destructive analyses of iron-polyphenolic complexes for reconstructing iron-gall inks historical recipes
Source: NPJ Herit Sci. 2025 Jul 28;13(1):371. doi: 10.1038/s40494-025-01946-x (PMC12303836; doi:10.1038/s40494-025-01946-x)
Supplement: Supplementary file 1 — Supplementary materials [file 40494_2025_1946_MOESM1_ESM.pdf]

Supporting Materials

T1.1 pH determination of commercial tannins and oak-gall extracts

Results of the triplicate measurements of the pH of commercial tannins solutions having a concentration of about 1 mg/mL and of the non-diluted oak-galls extracts.

Tannin	Conc. (mg/mL)	pH #1	pH #2	pH #3	Average	SD
QBC_S	1.04	4.86	4.86	4.82	4.847	0.019
GC_S	1.00	4.13	4.09	4.05	4.090	0.033
C_S	1.02	4.03	4.01	4.01	4.017	0.009
O10R	1.06	3.79	3.79	3.79	3.790	0.000
OE_FGL	1.02	3.96	3.99	3.94	3.963	0.021
T80_S	1.07	3.38	3.37	3.36	3.370	0.008
TG_L	1.01	3.82	3.98	3.98	3.927	0.075
T02	1.10	4.18	4.12	4.11	4.137	0.031

Exctract	pH #1	pH #2	pH #3	Average	SD
OG-Ex	3.50	3.48	3.51	3.50	0.02
OGWW-Ex	2.97	3.12	2.99	3.03	0.08
OGRW-Ex	3.49	3.51	3.49	3.50	0.01
OGP-Ex	2.27	2.25	2.23	2.25	0.02

T1.2. Total phenolic content determination via Folin-Ciacalteu assay

T1.2.1 GA calibration curve results.

The spectrophotometer used was configured to automatically zero the absorbance of the blank. Absorbance measurements were recorded as single readings at a wavelength of 765 nm (A_{765}).

GA Conc (ppm)	A_{765} (Intensity)
0	0
4	0.042
6	0.069
8	0.095
10	0.123
20	0.242

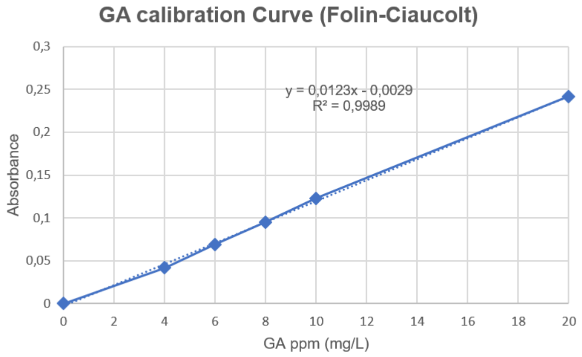

T1.2.2 Results of the triplicate A_{765} determination in commercial tannins and oak-gall extracts.

The Folin-Ciocalteu spectrophotometric assay, while widely used, is limited in accuracy due to both instrumental constraints and, more critically, the inherent complexity of natural matrices. For this reason, these values have to be considered as estimations of the total phenolic content of the sample.

Sample	Conc. (mg/mL)	dilution	A_{765} #1	A_{765} #2	A_{765} #3	Average mg GA eq.	mg of GA eq. per 100 mg	SD
QBC_S	1.070	100	0.105	0.106	0.112	898.92	84.01	2.34
GC_S	1.087	100	0.146	0.142	0.148	1205.15	110.87	1.87
C_S	1.034	100	0.089	0.088	0.088	741.73	71.73	0.37
O10R	1.084	100	0.105	0.100	0.106	866.40	79.93	1.97
OE_FGL	1.084	100	0.076	0.074	0.078	641.46	59.18	1.22
T80_S	1.089	100	0.150	0.146	0.150	1232.25	113.15	1.41

TG_L	1.036	100	0.106	0.105	0.108	888.08	85.72	0.98
T02	1.059	100	0.131	0.133	0.131	1094.04	103.31	0.72

Sample	dilution	A_{765} #1	A_{765} #2	A_{765} #3	Average mg GA eq.	mg of GA eq. per 1 mL Ex	SD
OG-Ex	50	0.113	0.121	0.116	486.04	48.60	1.34
OGWW-Ex	50	0.082	0.074	0.078	328.86	32.89	0.51
OGRW-Ex	50	0.079	0.082	0.081	339.70	33.97	1.33
OGP-Ex	50	0.051	0.054	0.053	225.88	22.59	0.51

T1.3 Results of the triplicate A_{530} determination in commercial tannins and oak-gall extracts (BuOH/HCl spectrophotometric assay).

In accordance with Zhen et al. (REF), the cyanidin equivalent (mg Cya eq.) was calculated in the case of commercial tannins solutions using the following equation:

$\frac{mg\ Cya\ eq.}{100\ mg\ sample} = \frac{A_{530}*d*V*MW*1000}{\varepsilon_{Cya}*l*m} * 100$where $\boldsymbol{A_{530}}$ is the absorbance read at 530 nm, $\boldsymbol{d}$ is the dilution factor (100 for all the tested samples), $\boldsymbol{V}$ is the stock solution volume (0.1 L for all samples), $\boldsymbol{MW}$ is the molecular weight of cyanidin (287.24 g/mol), $\boldsymbol{\varepsilon_{Cya}}$ is the molar extinction coefficient of cyanidin (34700 $Lmol^{-1}cm^{-1}$), $\boldsymbol{l}$ is the optical path (1cm), and finally, $\boldsymbol{m}$ is the mass of tannin.

In the case of OG extracts, the equation reported before would be simplified as follows:

$$\frac{mg\ Cya\ eq.}{1mL\ of\ Ex} = \frac{A_{530} * d * V * MW * 1000}{\varepsilon_{Cya} * l}$$

Sample	Conc. (mg/mL)	A_{530} #1	A_{530} #2	A_{530} #3	Average mg Cya eq.	mg of Cya eq. per 100 mg	SD
QBC_S	1.070	0.713	0.722	0.688	58.58	54.750	1.363
GC_S	1.087	0.008	0.005	0.003	0.441	0.406	0.192
C_S	1.034	0.029	0.030	0.025	2.318	2.242	0.212
O10R	1.084	1.446	1.443	1.392	118.124	108.971	2.317
OE_FGL	1.084	0.038	0.038	0.038	3.146	2.902	0.000
T80_S	1.089	0.015	0.015	0.015	1.242	1.140	0.000
TG_L	1.036	0.016	0.017	0.015	1.324	1.278	0.080
T02	1.059	0.003	0.000	0.003	0.166	0.156	0.135

Sample	A_{530} #1	A_{530} #2	A_{530} #3	Average mg Cya eq. per 1 mL	SD
OG-Ex	0.009	0.006	0.002	0.469	0.291
OGWW-Ex	0.007	0.005	0.004	0.441	0.126
OGRW-Ex	0.058	0.057	0.053	4.636	0.219
OGP-Ex	0.004	0.007	0.008	0.524	0.172

F.1 Infrared Spectral Changes in Commercial Tannins Induced by Iron Complexation

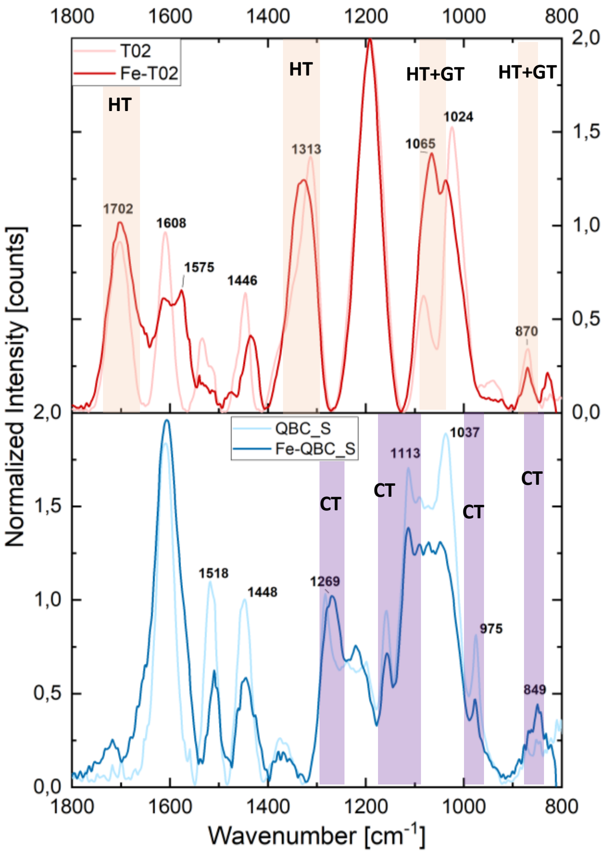

ATR-FTIR spectra of the commercial tannins T02 (GT) and QBC_S (CT) and their corresponding iron complexes. Each spectrum shown is an average of 5 to 6 selected spectra per pellet, chosen from a total of 1024 spectra collected using the FPA detector. Pellets were prepared in triplicate. The spectra illustrate the spectral changes induced by iron complexation in two representative tannins. Notably, the main GT markers in T02 (bands at ~1700 cm^{-1}, 1315 cm^{-1}, 1070 cm^{-1}, and 870 cm^{-1}) remain clearly identifiable in its iron-complexed form. Similarly, the characteristic CT markers in QBC_S (bands at ~1270 cm^{-1}, 1160 cm^{-1}, 1115 cm^{-1}, 975 cm^{-1}, 845 cm^{-1}, and the absence of the ~1700 cm^{-1} band) are still distinguishable in Fe-QBC_S. HT and GT markers are reported highlighted in orange, while CT markers in purple.

F.2 CW-EPR spectra of iron-commercial tannin complexes

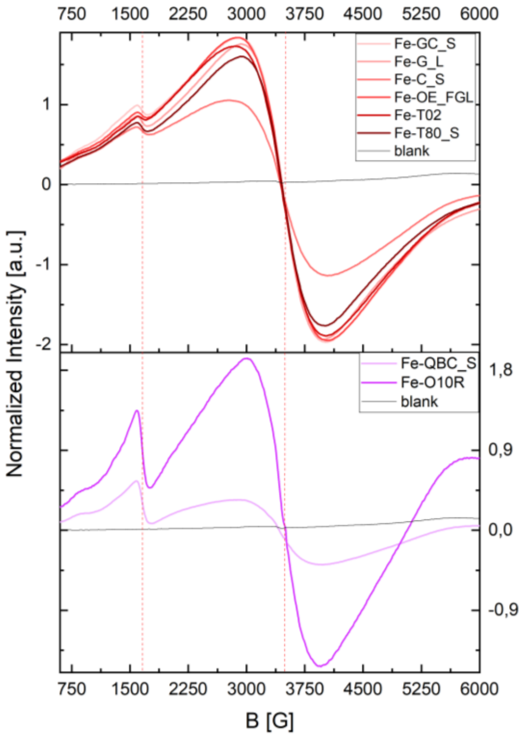

CW-EPR spectra of iron-commercial tannins. The spectra shown here undergone just the normalization by sample weight, enabling to observe the differences in the main signal intensities related to the abundance of paramagnetic centre per unit of weight. The spectrum of the quartz EPR tube is also reported (blank).

F.3 Variability of ATR-FTIR spectra of Fe complexes prepared from OG extracts

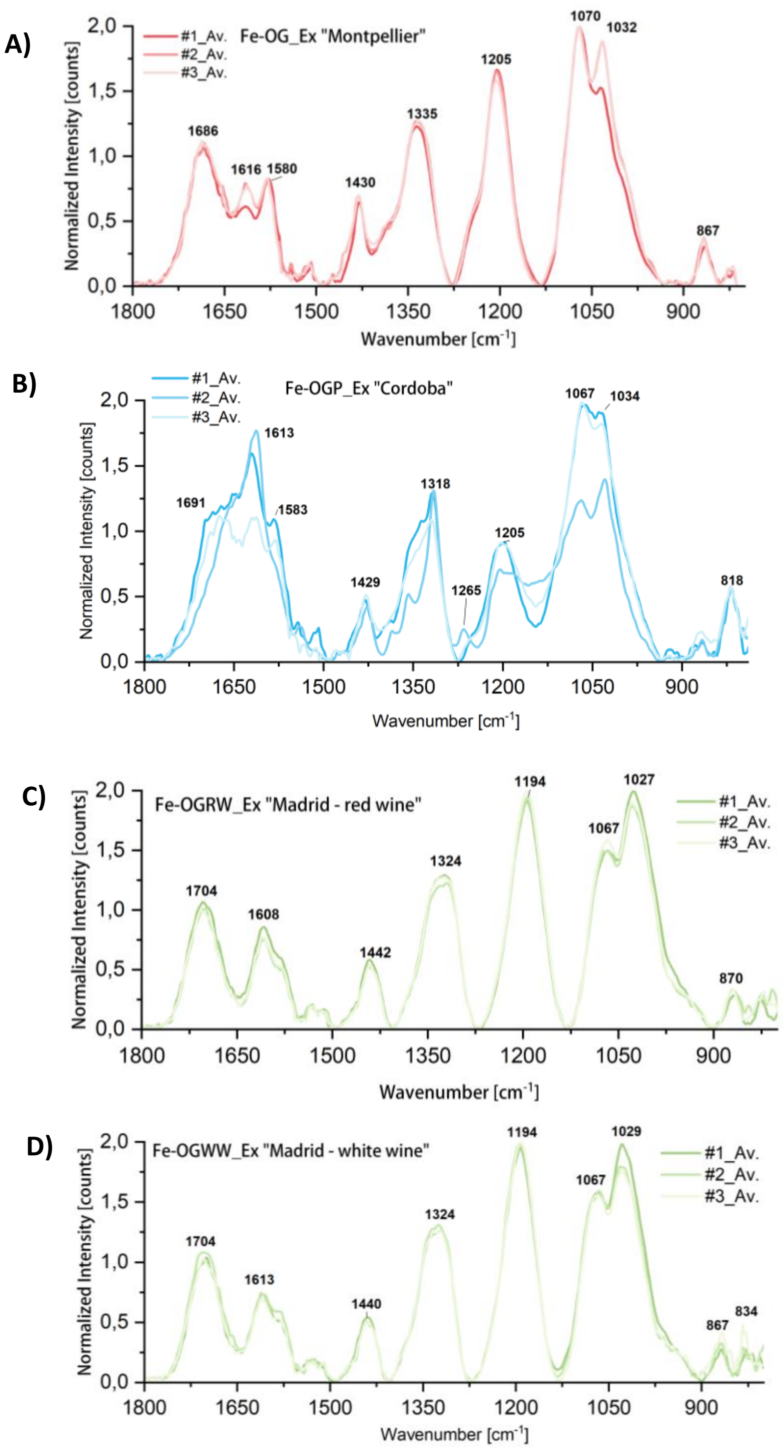

A-D) ATR-FTIR spectra of iron complexes prepared with OG extracts. Each spectrum represents the average of 5 measurements acquired on a single pellet (#n indicates the pellet number). The reported spectra highlight the variability within the spectral range of 1800–800 cm^{-1}.

The greatest variability was observed in the spectra of Fe-OGP (Fig. B), whereas in the other cases, only minor differences were noted, primarily in signal intensity or slight band shifts.

F.4 CW-EPR spectra of Fe complexes of OG extracts

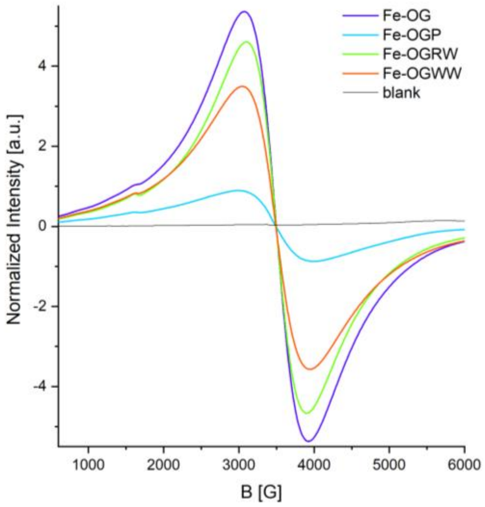

CW-EPR spectra of iron complexes formed with laboratory-prepared OG extracts. As in the case of the spectra reported in F.2, the spectra shown here undergone just the normalization by sample weight. The spectrum of the quartz EPR tube is also reported (blank).

F.5 Codex 1856 analysed fragments

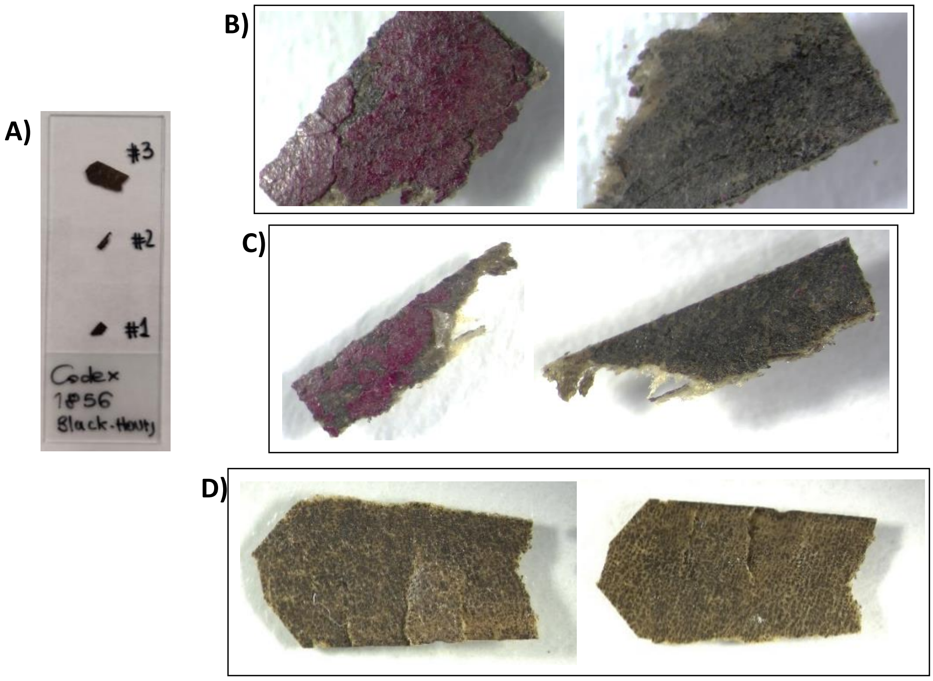

A) Image of the Black Hours fragments analysed. These fragments naturally detached from the manuscript's pages, and no precise information is available regarding their original folio of origin. B) Stereomicroscope image (5×) showing both sides of Sample 1. A vivid red-coloured layer is visible on the surface of the inked parchment. Since the characterization of this layer falls outside the scope of the current study, analyses were primarily carried out on the reverse side of the sample, where the coloured layer is absent. C) Stereomicroscope image (3.2×) of both sides of Sample 2. As observed in Sample 1, a red-coloured layer is present on one side of this sample too. Accordingly, the characterization was mainly conducted on the opposite side. D) Stereomicroscope image (1.25×) of both sides of Sample 3. In this case, the surface appears more homogeneous, though heavily degraded.
